# Transcriptome responses of an ungrafted Phytophthora root rot tolerant avocado (*Persea americana*) rootstock to flooding and *Phytophthora cinnamomi*

**DOI:** 10.1186/s12870-016-0893-2

**Published:** 2016-09-22

**Authors:** B. J. Reeksting, N. A. Olivier, N. van den Berg

**Affiliations:** 1 Department of Genetics, University of Pretoria, Pretoria, South Africa; 2 Department of Plant Science, University of Pretoria, Pretoria, South Africa; 3 Department of Microbiology and Plant Pathology, University of Pretoria, Pretoria, South Africa; 4 Forestry and Agricultural Biotechnology Institute, University of Pretoria, Pretoria, South Africa

**Keywords:** Avocado, Microarray, Hypoxia, Aquaporins, Glycolysis

## Abstract

**Background:**

Avocado (*Persea americana* Mill.) is a commercially important fruit crop worldwide. A major limitation to production is the oomycete *Phytophthora cinnamomi*, which causes root rot leading to branch-dieback and tree death. The decline of orchards infected with *P. cinnamomi* occurs much faster when exposed to flooding, even if flooding is only transient. Flooding is a multifactorial stress compromised of several individual stresses, making breeding and selection for tolerant varieties challenging. With more plantations occurring in marginal areas, with imperfect irrigation and drainage, understanding the response of avocado to these stresses will be important for the industry.

**Results:**

Maintenance of energy production was found to be central in the response to flooding, as seen by up-regulation of transcripts related to glycolysis and induction of transcripts related to ethanolic fermentation. Energy-intensive processes were generally down-regulated, as evidenced by repression of transcripts related to processes such as secondary cell-wall biosynthesis as well as defence-related transcripts. Aquaporins were found to be down-regulated in avocado roots exposed to flooding, indicating reduced water-uptake under these conditions.

**Conclusions:**

The transcriptomic response of avocado to flooding and *P. cinnamomi* was investigated utilizing microarray analysis. Differences in the transcriptome caused by the presence of the pathogen were minor compared to transcriptomic perturbations caused by flooding. The transcriptomic response of avocado to flooding reveals a response to flooding that is conserved in several species. This data could provide key information that could be used to improve selection of stress tolerant rootstocks in the avocado industry.

**Electronic supplementary material:**

The online version of this article (doi:10.1186/s12870-016-0893-2) contains supplementary material, which is available to authorized users.

## Background

Flooding is a complex stress which can be caused by natural floods, high rainfall, over-irrigation, or perched water tables [1]. It comprises several individual stresses, including hypoxia, changes in soil pH, and increased pathogen activity, all of which contribute to the overall stress experienced by the plant [2]. This leads to reduced photosynthesis, stomatal closure and decline in root hydraulic conductivity, causing a reduction in growth and yield [3, 4]. The multifactorial nature of flooding stress makes development of tolerant varieties of commercially important crops challenging [5]. In general, naturally flood-tolerant plants either utilize a mechanism that results in re-aeration of flooded tissue, or they conserve energy in order to resume growth once flooding has subsided [6–8]. Hypoxia is the main constraint for normal plant growth under flooding conditions. Reduced oxygen availability limits mitochondrial respiration, leading to a higher demand of ATP generated through glycolysis [4, 9]. Under these conditions fermentation allows the regeneration of NAD^+^ in order to maintain glycolysis, and thus energy production. However, the ATP produced via fermentation (2 mol ATP per mol glucose) is much less than that produced by mitochondrial respiration (38 mol ATP per mol glucose). This leads to an energy shortage in the plant, requiring much higher levels of fermentation in order to maintain the energy balance [10]. Indeed, higher induction of genes encoding glycolytic enzymes has been seen in several species in response to flooding, including *Arabidopsis*, rice and poplar [11]. This induction was paralled by repression of transcripts related to mitochondrial respiration. Increased glycolytic flux represents a compensation mechanism in order to increase ATP production under oxygen-limited conditions [12]. However, this increased strain on glycolysis and fermentation can lead to a heightened demand for carbohydrates and depletion of reserves (‘Pasteur effect’) [4]. This is exacerbated by the decline in photosynthesis under these conditions [13]. The ability of plants to maintain the energy balance via fermentation is characteristic of flood-tolerant plants [4].

Stomatal conductance has been seen to decline in several species during flooding and reduced hydraulic conductivity is thought to contribute to these declines as root water uptake and subsequent transport to the shoots is affected by flooding [14]. These reductions are often an early response [15] and are generally more pronounced in flood-susceptible plants. Reduced root hydraulic conductance, which is highly dependent on environmental conditions [16], can result from damage to roots or decreased activity or reduced expression of aquaporins, the water channel membrane proteins [4, 17]. These are multifunctional proteins that transport water, gases, boron, silicon and reactive oxygen species (ROS) [16]. These proteins facilitate uptake of soil water and can contribute substantially (>60 %) to the root hydraulic conductivity [16, 17] and reduced expression of these genes may limit aquaporin function [18]. Disruption of water uptake can have adverse effects on the growth and survival of plants during flooding.

Avocado (*Persea americana* Mill.) is grown worldwide for its oil-rich fruit. However, it is susceptible to flooding [19, 20], with even transient flooding causing severe damage. The largest threat to avocado production is caused by the oomycete pathogen *Phytophthora cinnamomi* Rands. This pathogen attacks the feeder roots of avocado plants causing root rot (PRR), leading to branch-dieback and eventual tree death. Flooding exacerbates the disease progression of PRR, causing faster decline of trees and greater losses in production [21, 22]. Currently, commercial producers utilize PRR tolerant rootstocks which are grafted with commercial scions in order to minimize damage caused by *P. cinnamomi*. Whilst these rootstocks are effective in well-drained soils, they do not necesarily perform well in areas that experience transient flooding. Tolerance to flooding appears to be determined by the rootstock, and not the scion [23, 24]. Selection of rootstocks that exhibit tolerance to both flooding and *P. cinnamomi* would greatly benefit the industry, however current selection programmes for PRR tolerance do not consider tolerance to flooding.

Molecular studies assessing the response of avocado to flooding are limited, with no large expression studies available. The aim of this study was to investigate the transcriptome response of a PRR tolerant rootstock previously shown to be susceptible to flooding. The effects of *P. cinnamomi* presence in the soil prior to flooding were also investigated to determine whether this would result in a faster decline of flooded avocado trees. In this work, a global analysis of gene expression was performed utilizing a custom avocado Agilent array.

## Methods

### Plant material and RNA isolation

One-year old clonal ‘Dusa™’ plantlets (Westfalia Technological Services, Tzaneen, South Africa) were grown in 2 L containers in a soil-perlite mix (1:1, v:v) in a glasshouse (average max temp. 24.9 °C, average min temp. 13.7 °C) at the Forestry and Agricultural Biotechnology Institute (25° 45' 19.63" S, 28° 14' 7.75"E, University of Pretoria, South Africa). Plants were watered 3–4 times weekly and supplemented with Hoagland’s solution [25] once a week. Treatments were split into four groups; control plants (C), infected plants (I), flooded plants (F), and plants exposed to a combination of flooding and infection (FI). The experiment was laid out in a randomized block design and three biological replicates per treatment, per time-point were taken where each biological replicate consisted of two plants. *Phytophthora cinnamomi* was isolated from commercial blocks of declining avocado orchards in Tzaneen, Limpopo, South Africa. Pre-trials assessing disease development were conducted to confirm pathogenicity of isolates. Inoculation with *P. cinnamomi* was carried out using a zoospore suspension 2.5 × 10^4^ zoospores/ml as reported previously [26]. Plants were flooded 7 days after infection in order to allow establishment of infection. Flooding was carried out by immersing plants in plastic reservoirs filled with tap water to 1 cm below potting medium. Infection was confirmed by re-isolation of the pathogen and subsequent use of the species specific LPV3 primers [27]. Root samples were harvested at six time-points relative to the start of flooding (0, 8, 22, 48, 96 h, and 7 days) and immediately stored at -80 °C. Total RNA was extracted and purified [26]. Total RNA concentration and integrity was estimated using the NanoDrop® ND-1000 (Nanodrop Technologies Inc., Montchanin, USA) spectrophotometer and non-denaturing 2 % TAE agarose gels as well as using the Bio-Rad Experion™ automated electrophoresis system (Bio-Rad, CA, USA).

### Microarray analysis

Transcripts from the *de novo* sequencing of the avocado root transcriptome in response to flooding and *P. cinnamomi* infection [26] were used for probe design. From these data, 6141 annotated contigs and 1987 singletons were selected for *Persea americana*. Bi-directional sequences for an additional 516 unannotated contigs were also included. Therefore a total of 9160 avocado transcripts, selected as described in Reeksting 2014, were used for probe design. The total number of unique transcripts represented on the array was 9625, which included 465 *P. cinnamomi* genes. These pathogen genes were selected based on their role in pathogenicity. Sequences were uploaded onto the Agilent eArray (https://earray.chem.agilent.com/earray/) website in FASTA format for probe design using the SurePrint HD format slides (8x15K) with 60mer oligonucleotides. Feature layout was randomized and empty features were filled with random duplicate probes. Agilent linker sequences were included at the 3’ end of each probe. A common reference pool was generated by pooling 2 μg of RNA from each treatment and time-point. Four to 6 μg of RNA was used for first strand cDNA synthesis. Single-stranded cDNA was synthesized according to manufacturer’s instructions using Superscript™ III Reverse transcriptase (Invitrogen) in a total volume of 30 μl. First strand synthesis was primed with random nonamer (N_9_, Inqaba Biotec, Sunnyside, South Africa) and oligo(dT) (dT_23_VN, Inqaba) primers. cDNA clean-up was carried out using an RNA clean-up kit (Qiagen RNeasy® MinElute™) to remove hydrolysed RNA. Concentration and purity of cDNA was determined using a Nanodrop® ND-1000. Samples were dried *in vacuo* (SpeediVac) at 50 °C. Pellets were re-dissolved in 100 mM NaHCO_3_ buffer (0.2 M Na_2_CO_3_, 0.2 M NaHCO_3_, pH 9.0) and incubated at 37 °C for 10 min. Samples were labelled with Cy5 and the reference was labelled with Cy3 (CyDye Post-Labeling Reactive Dye Pack, GE Healthcare Life Sciences). The reaction was terminated using 3 M NaOAc (pH 5.2). Excess dye was removed using the RNeasy® MinElute™ Clean-up kit (Qiagen) according to manufacturer’s instructions. Labelled cDNA was eluted in 30 μl RNase-free H_2_O water. The yield and specific activity was calculated and hybridization was carried out according to the two-colour microarray-based gene expression analysis protocol (Agilent). Microarray slides were scanned using the Axon GenePix 4000B scanner (Molecular Devices, CA, USA) and Axon GenePix 6.0 software (Molecular Devices) was used for image assessment. GenePix Array List (GAL) files were generated by Agilent and loaded into GenePix to link information of each printed spot to analyze results. Following automated spot detection using the software, manual feature alignment was performed to validate spot finding. Flagging of features was based on saturation and signal-to-noise ratios (SNR). Features with SNR < 2 in both channels were excluded from further analysis, as were features with foreground saturation > 20 %. The dataset from this study are available from the NCBI’s Gene Expression Omnibus through GEO Series accession number GSE81297 (http://www.ncbi.nlm.nih.gov/geo/query/acc.cgi?acc=GSE81297) according to MIAME guidelines.

### Statistical analysis

The LIMMA (Linear models for microarray data, www.bioconductor.com) package was used in the R version 3.1.0 environment (R Foundation for Statistical Computing, http://www.R-project.org) to perform statistical analysis of microarray data. Background correction was performed using the ‘normexp’ function in LIMMA using an offset of 50 [28]. Within-array normalization was carried out using Robust Spline normalization. Gquantile normalisation was used to normalize between arrays. Fold changes and standard errors were estimated by fitting a linear model for each gene in LIMMA (lmFit). Empirical Bayes smoothing was applied to the standard errors (eBayes). Finally, *P*-values were adjusted for multiple testing using the false discovery rate (FDR) correction. A standard pair-wise Pearson correlation (*r*) was performed using normalized M-values to determine concordance between biological replicates. Targets were defined as differentially expressed if the log_2_ ratio was greater or equal to 1 or smaller or equal to -1 (log_2_ ratio ≥ 1 and log_2_ ratio ≤ -1) and the adjusted *P*-value was less than or equal to 0.05 (*P* ≤ 0.05).

### Functional annotation, clustering, and pathway analysis

Functional annotation of targets on the microarray was performed utilizing the desktop cDNA Annotation System (dCAS) software (v 1.4.3) [29]. Gene ontology (GO) terms describing biological processes, molecular functions and cellular components were assigned using Blast2GO software (B2G; http://www.blast2go.com). Default parameters were used with a cut-off FDR of 0.05. Venn diagrams were drawn using Venny (http://bioinfogp.cnb.csic.es/tools/venny/index.html). Hierarchical clustering, by average linkage, was performed using Multi Experiment Viewer (MeV) version 4.8.1 [30].

### RT-qPCR

Validation of gene expression levels obtained from the microarray analysis was performed using RT-qPCR. Single-stranded cDNA was synthesized using the ImProm-II™ single strand cDNA system according to manufacturer’s instructions (Promega Corporation, Madison, USA). Random hexamers (0.5 μg, Invitrogen Life Technologies, California, USA) were used to prime first strand synthesis. The intron-spanning flavone-3-hydroxylase (F3H) primers, F3H F 5’-TCTGATTTCGGAGATGACTCGC-3’ and F3H R 5’-TGTAGACTTGGGCCACCTCTTT-3’ (Inqaba Biotec) were used to assess genomic DNA (gDNA) contamination. The expression of nine avocado genes was investigated. Three endogenous control genes (*Actin*, *18S*, *Alpha*-*1 tubulin*) were used for normalisation. Primer sequences and annealing temperatures for endogenous control genes and the nine avocado genes are presented in Additional file 1: Table S1. PerlPrimer v1.1.21 (http://perlprimer.sourceforge.net) was used for primer design and primers were synthesized by Integrated DNA Technologies (IA, USA). Specificity of primers was initially tested by conventional PCR and confirmed by the presence of a single melting curve during RT-qPCR. Optimum dilutions to use were determined by generation of standard curves (1:5, 1:10, 1:20, 1:50, 1:100, 1:500, 1:1000) for each primer set. Reactions were set up in a 96-well plate and RT-qPCR was performed using the Bio-Rad CFX96 ™ Real Time PCR Detection System using Sensimix™ SYBR No-ROX (Bioline Ltd, London, UK). Three biological reps for each treatment were included for each time-point, and all reactions were performed in triplicate. Data analysis was performed using the Bio-Rad CFX Manager Software. Statistical significance of the data was determined by one-way ANOVA followed by a Student’s *t*-test carried out with JMP® version 10.0.0 software (http://www.jmp.com/, SAS Institute, Inc.). Significance was assessed at *P* < 0.05. Data were graphed in GraphPad Prism® version 6.03 (www.graphpad.com).

## Results and discussion

### Transcriptome responses of avocado

Microarray analysis was performed using root samples taken at 22 and 48 h post-flooding (hpf) and results were validated using RT-qPCR (Additional file 2: Table S2). Flooding was found to have a profound impact on the transcriptome of avocado, causing the induction of more than 1000 transcripts (Log_2_FC > 1, Adj. *P*-value < 0.05), with a similar number repressed (Table 1). Comparison of flooded treatments that were infected (FI) to those that were not infected (F) yielded no significant changes in gene expression between the two treatments. This suggests that the more subtle transcriptome changes that occur in response to infection may be masked by the response to flooding. This is supported by differences in transcript expression seen between infected (I) and uninfected (C) plants that were not subjected to flooding. There were only small differences in gene expression at 22 h post-flooding (hpf, 8 days post-infection) between infected (I) and control (C) plants that were not flooded (Table 1). Induced transcripts included transcripts homologous to *alcohol dehydrogenase* from *Streptomyces* sp. (Pa_Sin_GI32N0T02IWOXV, log_2_FC = 1.23), *beta*-*1*,*3*-*glucanase* (Pa_Contig00542, log_2_FC = 1.95296) from *Vitis vinifera*, and a contig with no significant homology to any known sequence (Pa_NA_RC_Contig07628, log_2_FC = 2.46). Several defence-related transcripts were found to be up-regulated at 48 hpf in response to infection (I), including *β*-*1*,*3*-*glucanases*, *cytochrome P450* (Pa_Contig07667), *chitotriosidase* (Pa_Contig00472), *chitinase* (Pa_Contig01014), *pathogen*-*related protein*-*like* (Pa_Contig01063), *GDSL esterase*/*lipase* (Pa_Contig00520) and *germin*-*like proteins* (Additional file 3: Table S3). β-1,3-glucanases are known pathogenesis-related proteins [31]. Pa_Contig00542, a *beta*-*1*,*3*-*glucanase*, seen to be induced at 22 hpf, was also up-regulated (log_2_FC = 1.72) at 48 hpf (9 days post-infection). *Cytochrome P450* has been shown to play a role in plant defence [32], *chitoriosidase* is a chitinase and these proteins also belong to the pathogenesis-related proteins [33], GDSL esterase/lipase has been described to play a role in the regulation of plant development, secondary metabolite synthesis, and defence response [34], and germin-like proteins are involved in plant development and defence [35].

**Table 1:** Summary of the differentially expressed avocado transcripts^a^

Comparison	FI vs C	I vs C	F vs C	FI vs I	F vs I	F vs FI
22 h						
Up-regulated	1134	4	1010	1057	954	0
Down-regulated	779	0	769	795	814	0
Ratio	1.46	na	1.31	1.33	1.17	na
*r*	−0.43	0.89	−0.38	−0.39	−0.38	0.90
48 h						
Up-regulated	1217	16	1230	1129	0	1200
Down-regulated	1068	10	1000	1047	1	1006
Ratio	1.14	1.6	1.23	1.08	0	1.19
*r*	−0.55	0.86	−0.55	−0.48	0.90	−0.51
	22 vs 48 h (FI)	22 vs 48 h (I)	22 vs 48 h (C)	22 vs 48 h (F)		
Up-regulated	45	30	0	7		
Down-regulated	25	32	8	11		
Ratio	1.8	0.94	0	0.64		
*r*	0.80	0.82	0.90	0.82		

^a^Any duplicate probes were removed before calculating the number of significantly expressed transcripts. Comparisons are separated into time-points, with the number of up-regulated and down-regulated transcripts (Adj. *P*-value <0.05, log_2_fold change >1 or < -1) shown for each comparison. Comparisons between time-points are also shown. The ratio of up-regulated to down-regulated genes is indicated. The Pearson correlation coefficient (*r*) is indicated for each comparison. Flooded and infected (FI), infected (I), flooded (F), and control (C)

Pearson correlations assessing the expression patterns between comparisons indicated strong similarities in expression patterns between plants that were flooded (FI and F) and between plants that were not flooded (C and I). Indeed, there were no significantly differentially expressed genes between FI and F at 22 hpf. The ratio of up-regulated to down-regulated transcripts in response to flooding was generally higher at 22 hpf than at the later time-point, indicating a higher number of repressed genes as flooding continues. Strong positive correlations in expression were also seen across time-points for the same treatment, whilst negative correlations were seen when comparing flooded to non-flooded treatments (Table 1).

**Fig. 1:**
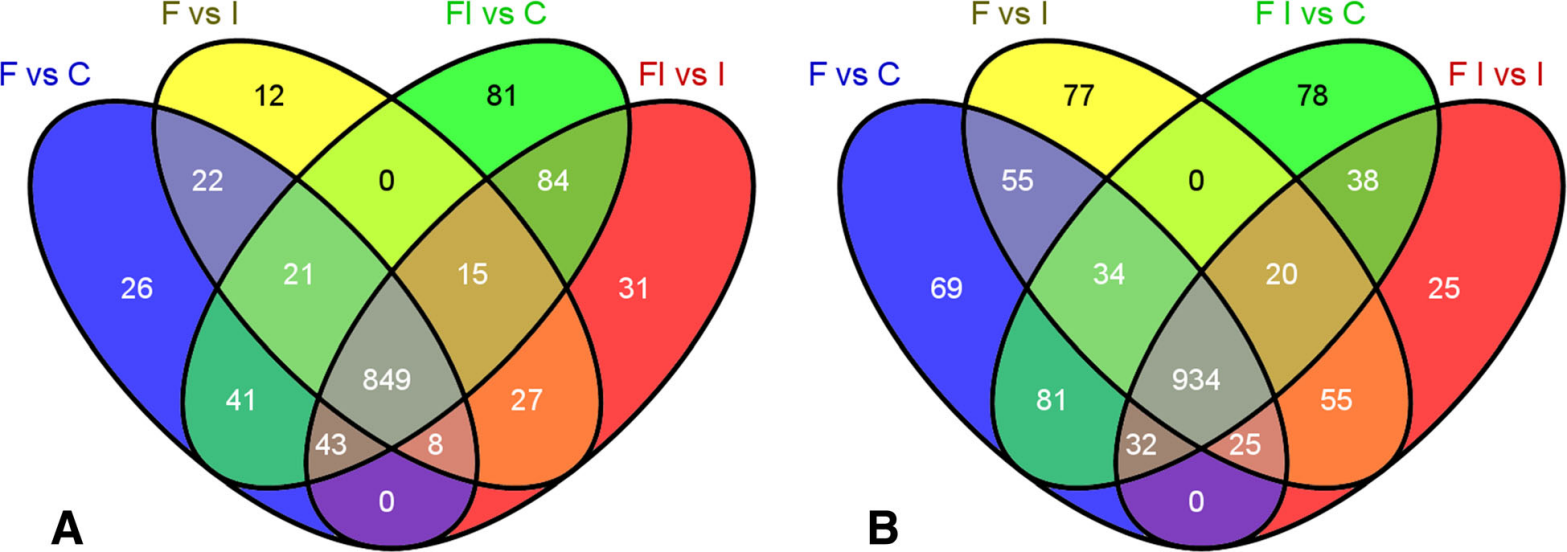
Comparison of the induced avocado transcripts in flooded to non-flooded treatments at 22 h post-flooding (**a**) and 48 h post-flooding (**b**) Values for transcripts with more than one probe present on the array were first averaged and then subjected to the thresholds to determine differential expression

**Fig. 2:**
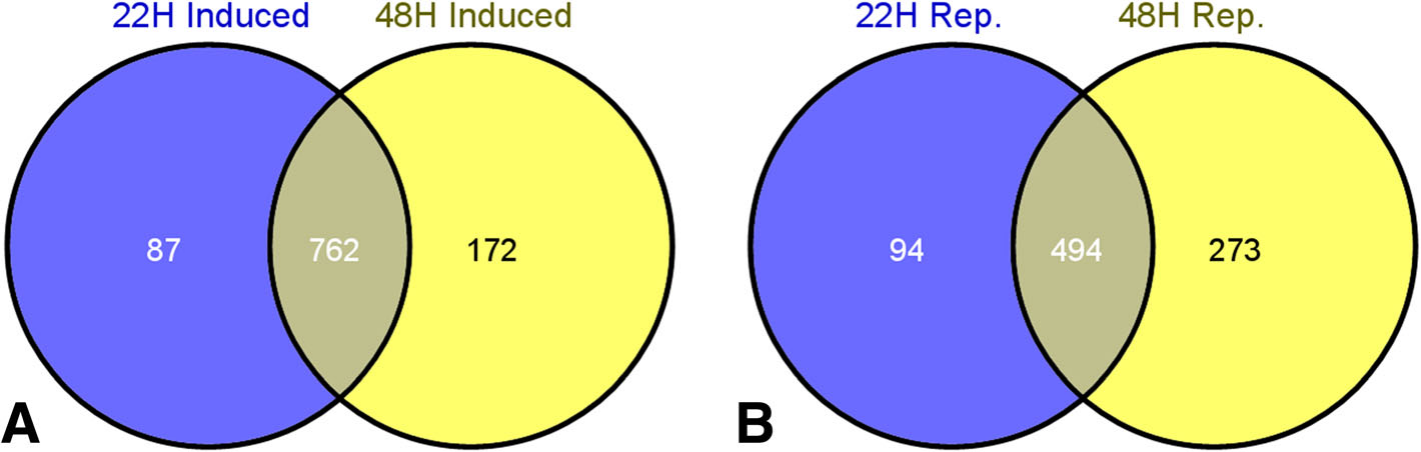
Venn diagrams illustrating the transcripts that are commonly induced (**a**) and repressed (**b**) in all flooding treatments at both 22 h post-flooding and 48 h post-flooding. Shared transcripts are illustrated where the two circles meet whilst unique time-point specific genes are shown in only one of the two circles

The majority of differentially expressed transcripts (900) that were induced in response to flooding were shared between infected and uninfected treatments (Fig. 1). This indicates a conserved response to flooding, regardless of the presence or absence of pathogen. A similar trend was seen in transcripts that were repressed (Additional file 4: Figure S1). Although there were some time-point specific genes, many of the genes whose expression was altered at 22 hpf maintained this change at 48 hpf (Fig. 2).

**Fig. 3:**
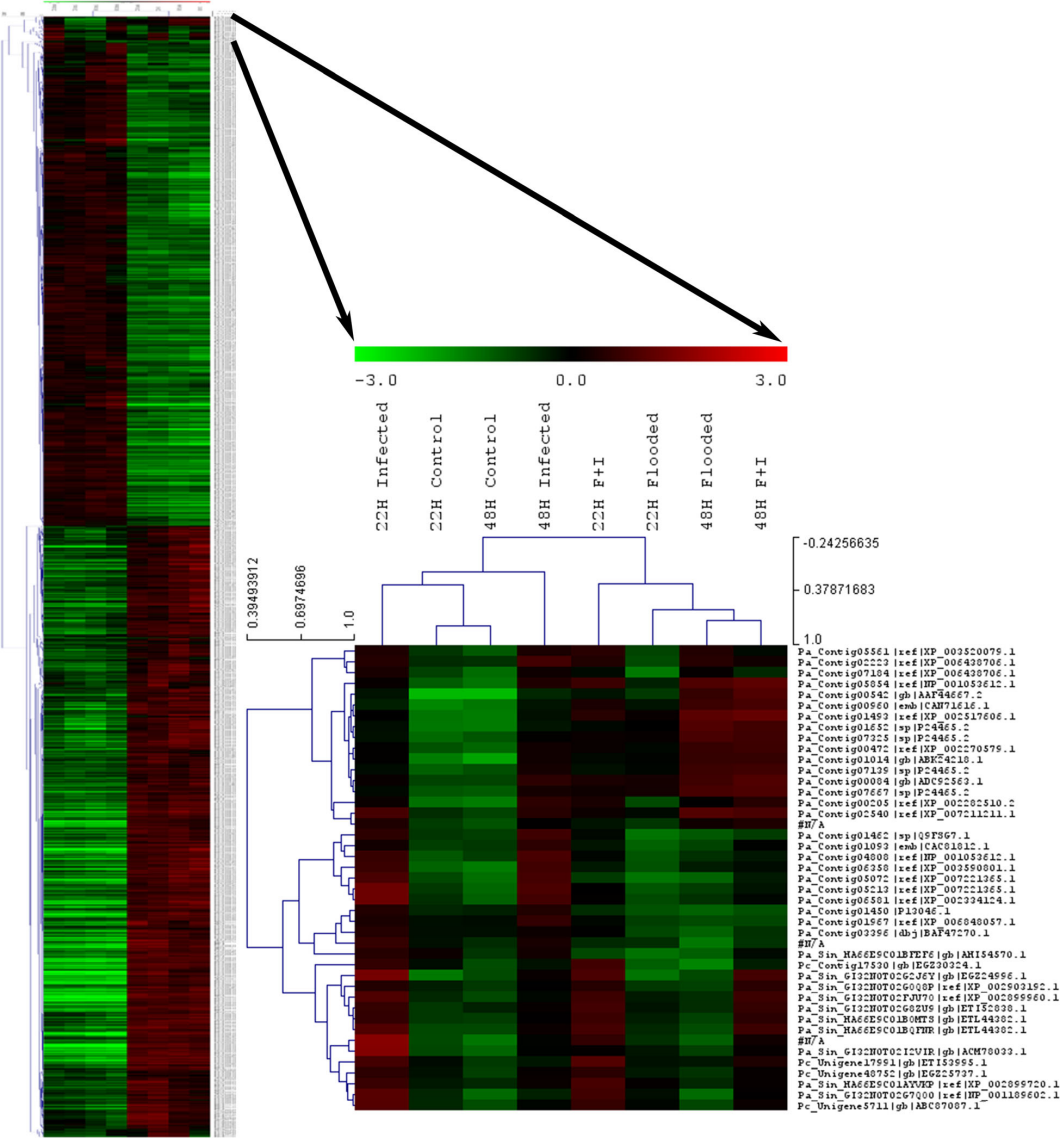
HCL performed on all avocado transcripts found to be commonly induced or repressed in all flooded treatments that showed significant fold changes in individual comparisons. The enlarged cluster represents transcripts differentially expressed at different time-points and based on pathogen presence. The colour-scale indicates log_2_FC and the branches of the trees are ordered according to the Pearson correlation coefficient (*r*)

Hierarchical clustering (HCL) using average-linkage was performed on all transcripts significantly induced or repressed in all flooded relative to non-flooded comparisons (Fig. 3). Flooded treatments showed similar expression patterns to one another whilst non-flooded treatments exhibited similar expression patterns, regardless of whether they were infected or not. A strong negative correlation (-0.71, Pearson) was evident between flooded and non-flooded treatments, indicating large transcriptomic changes induced by flooding stress. This can be seen clearly in Fig. 3, where the non-flooded treatments (on the left) show distinctly opposing patterns of gene expression from flooded treatments (on the right). These two groups were subsequently divided by time-point, with infection status only causing subtle transcriptomic changes between treatments. Differences caused by the presence of the pathogen were more conspicuous in non-flooded treatments (Fig. 3).

### Effects on carbon metabolism

Enrichment analysis was performed for transcripts showing significant (log_2_FC > 1 or < -1, adj. *P*-value < 0.05) differential expression. Many of the categories that were over-represented for upregulated transcripts at 22 hpf in flooded to non-flooded comparisons were related to the response to hypoxia (Additional file 5: Figure S2). This included cofactor binding, coenzyme binding, oxidoreductase activity, oxidation-reduction processes, glycolysis, dioxygenase activity, and response to hypoxia. Transferase activity and oxidation-reduction processes were the most represented categories for upregulated transcripts of all comparisons at 48 h (Additional file 6: Figure S3). More than 23 % of the sequences were associated with transferase activity. Other categories that were over-represented in flooded treatments were generally involved in enzyme function, such as ‘cofactor binding’ or other energy-related processes [36], such as oxidoreductases which catalyse electron transfer and are likely involved in regeneration of NAD^+^ from NADH under flooded conditions in order to drive further glycolytic reactions. Dioxygenase activity and response to hypoxia were also enriched categories in flooded plants and have a wide range of biological roles. It has been suggested that stress-induced haemoglobins may function as dioxygenases, detoxifying nitric oxide (NO) produced during hypoxia [37]. Calcium ion binding was also over-represented in certain comparisons (FI vs C) and calcium has been suggested to have a critical role in signalling oxygen stress within the plant and has been found to be required for the induction of *alcohol dehydrogenase* in maize and *Arabidopsis* [38].

Increased glycolytic flux [17], coupled to increased expression of glycolysis-related genes, occurs in hypoxic conditions in order to compensate for decreased ATP production associated with inhibited respiration [12]. Induction of glycolytic enzymes was seen in avocado in response to flooding, where nine of the 10 enzymes in the glycolytic pathway showed increases in expression. These included *hexokinase*, *phosphoglucose isomerase*, *phosphofructose kinase*, *fructose*-*bisphosphate aldolase*, *triosephosphate isomerase*, *glyceraldehyde 3*-*phosphate dehydrogenase*, *phosphoglycerate kinase*, *enolase*, and *pyruvate kinase* (Table 2). Only *phosphoglycerate mutase* did not show significant increases in expression. Induction of glycolytic genes has been seen in several species in response to flooding and comparisons of two oak varieties differing in tolerance to flooding revealed stronger induction of glycolytic genes in the more flood-tolerant variety [39].

**Table 2:** Glycolytic enzymes differentially expressed in avocado in response to flooding

Name	Annotation	E-value	FI vs C		F vs C	
			FC 22 h	FC 48 h	FC 22 h	FC 48 h
Pa_Contig00049	*PREDICTED*: *Vitis vinifera hexokinase*-*1*-*like* (*LOC100242358*). *mRNA*	0	3.06	3.93	3.47	4.38
Pa_Contig00073	*PREDICTED*: *Nelumbo nucifera pyruvate kinase*, *cytosolic isozyme* (*LOC104591283*), *transcript variant X4*, *mRNA*	0	2.18	2.22	1.99	2.19
Pa_Contig00092	*PREDICTED*: *Nelumbo nucifera pyruvate kinase*, *cytosolic isozyme*-*like* (*LOC104612526*), *mRNA*	0	2.67	2.44	2.31	1.98
Pa_Contig00101	*PREDICTED*: *Elaeis guineensis 2*,*3*-*bisphosphoglycerate*-*independent phosphoglycerate mutase* (*LOC105046041*), *mRNA*	0	2.35	1.65	2.05	1.77
Pa_Contig00105	*PREDICTED*: *Nelumbo nucifera pyruvate kinase*, *cytosolic isozyme* (*LOC104610148*), *transcript variant X5*, *mRNA*	0	−2.35	−3.31	−2.77	−3.21
Pa_Contig00106	*PREDICTED*: *Nelumbo nucifera pyruvate kinase*, *cytosolic isozyme*-*like* (*LOC104612526*), *mRNA*	0	4.71	4.78	3.73	4.85
Pa_Contig00124	*Magnolia liliiflora GADPH mRNA for glycolytic glyceraldehyde*-*3*-*phosphate dehydrogenase*	0	6.4	5.54	5.61	5.38
Pa_Contig00281	*PREDICTED*: *Elaeis guineensis phosphoglycerate kinase*, *cytosolic* (*LOC105059872*), *transcript variant X2*, *mRNA*	0	3.6	3.41	2.99	3.04
Pa_Contig00331	*Persea americana mRNA for fructose*-*bisphosphate aldolase* (*alf gene*)	1e^-175^	2.66	3.00	3.22	2.90
Pa_Contig00355	*Persea americana mRNA for fructose*-*bisphosphate aldolase* (*alf gene*)	0	−1.6	−2.12	−1.56	−2.17
Pa_Contig00366	*PREDICTED*: *Phoenix dactylifera ATP*-*dependent 6*-*phosphofructokinase 3*-*like* (*LOC103714334*), *mRNA*	0	9.36	9.81	9.27	10.47
Pa_Contig00411	*PREDICTED*: *Vitis vinifera pyrophosphate*--*fructose 6*-*phosphate 1*-*phosphotransferase subunit alpha* (*LOC100249662*), *mRNA*	0	21.82	13.02	19.66	13.00
Pa_Contig00493	*PREDICTED*: *Nelumbo nucifera glucose*-*6*-*phosphate isomerase*, *cytosolic* (*LOC104595713*), *mRNA*	0	3.45	2.51	2.87	2.32
Pa_Contig00650	*Actinidia eriantha fructokinase mRNA*, *complete cds*	0	5.40	8.92	5.79	8.86
Pa_Contig00959	*PREDICTED*: *Nelumbo nucifera triosephosphate isomerase*, *cytosolic* (*LOC104602457*), *mRNA*	0	−1.87	−2.75	−2.29	−2.69
Pa_Contig01066	*M.liliiflora GADPH mRNA for glycolytic glyceraldehyde*-*3*-*phosphate dehydrogenase*	0	3.12	2.35	2.44	1.91
Pa_Contig01152	*PREDICTED*: *Nelumbo nucifera triosephosphate isomerase*, *cytosolic* (*LOC104602457*), *mRNA*	0	5.38	7.28	5.20	6.76
Pa_Contig01301	*PREDICTED*: *Nelumbo nucifera triosephosphate isomerase*, *chloroplastic*-*like* (*LOC104605564*), *transcript variant X3*, *misc*_*RNA*	0	−1.62	−1.87	−1.62	−2.01
Pa_Contig01521	*PREDICTED*: *Nelumbo nucifera NADP*-*dependent glyceraldehyde*-*3*-*phosphate dehydrogenase* (*LOC104603324*), *mRNA*	0	3.37	3.14	2.93	2.37
Pa_Contig01833	*PREDICTED*: *Nelumbo nucifera probable fructokinase*-*1* (*LOC104605215*), *mRNA*	1e^-150^	−1.98	−1.86	−1.73	−2.19
Pa_Contig02126	*PREDICTED*: *Phoenix dactylifera pyrophosphate*--*fructose 6*-*phosphate 1*-*phosphotransferase subunit beta* (*LOC103718504*), *mRNA*	3e^-117^	−1.86	−2.65	−1.74	−2.89
Pa_Contig02161	*PREDICTED*: *Vitis vinifera pyrophosphate*--*fructose 6*-*phosphate 1*-*phosphotransferase subunit beta* (*LOC100256839*), *mRNA*	0	−1.39	−2.36	−1.48	−2.04
Pa_Contig02414	*PREDICTED*: *Malus domestica hexokinase*-*1*-*like* (*LOC103449780*), *mRNA*	2e^-146^	ns	2.16		−1.67
Pa_Contig02512	*58* % *coverage*	3e^-56^	20.27	11.41	18.3	11.48
Pa_Contig02723	*PREDICTED*: *Nelumbo nucifera NADP*-*dependent glyceraldehyde*-*3*-*phosphate dehydrogenase*-*like* (*LOC104610711*), *mRNA*	0	2.94	2.89	2.83	2.57
Pa_Contig03335	*PREDICTED*: *Amborella trichopoda enolase* (*LOC18441538*), *mRNA*	4e^-137^	5.30	3.98	3.88	3.74
Pa_Contig03561	*PREDICTED*: *Nelumbo nucifera glyceraldehyde*-*3*-*phosphate dehydrogenase GAPCP2*, *chloroplastic*-*like* (*LOC104611012*), *mRNA*	3e^-75^	2.25	2.33	2.24	2.24
Pa_Contig04410	*PREDICTED*: *Phoenix dactylifera glucose*-*6*-*phosphate isomerase*, *cytosolic* (*LOC103702710*), *transcript variant X1*, *mRNA60* % *coverage*	1e^-67^	5.11	3.50	3.83	3.54
Pa_Contig04576	*Annona cherimola enolase mRNA*, *complete cds*	6e^-78^	3.83	3.82	3.16	3.65
Pa_Contig05176	*PREDICTED*: *Vitis vinifera phosphoglycerate mutase* (*LOC100245371*), *mRNA*	1e^-125^	−1.53	−1.57	−1.62	−1.59
Pa_Contig06531	*PREDICTED*: *Camelina sativa glyceraldehyde*-*3*-*phosphate dehydrogenase GAPC2*, *cytosolic* (*LOC104772375*), *mRNA*	1e^-54^	3.22	2.52	2.32	2.17
Pa_Contig07171	*PREDICTED*: *Nicotiana sylvestris glucose*-*6*-*phosphate isomerase*, *cytosolic* (*LOC104225311*), *transcript variant X3*, *mRNA*	2e^-14^	3.80	3.31	3.16	2.68
Pa_Contig07301	*PREDICTED*: *Phoenix dactylifera glyceraldehyde*-*3*-*phosphate dehydrogenase GAPCP2*, *chloroplastic*-*like* (*LOC103696512*), *mRNA 140 bp*	5e^-41^	−2.38	−1.62	−2.62	−1.75

Adj. *P*-value <0.05, log_2_fold change >1 or < -1 in at least one comparison

**Fig. 4:**
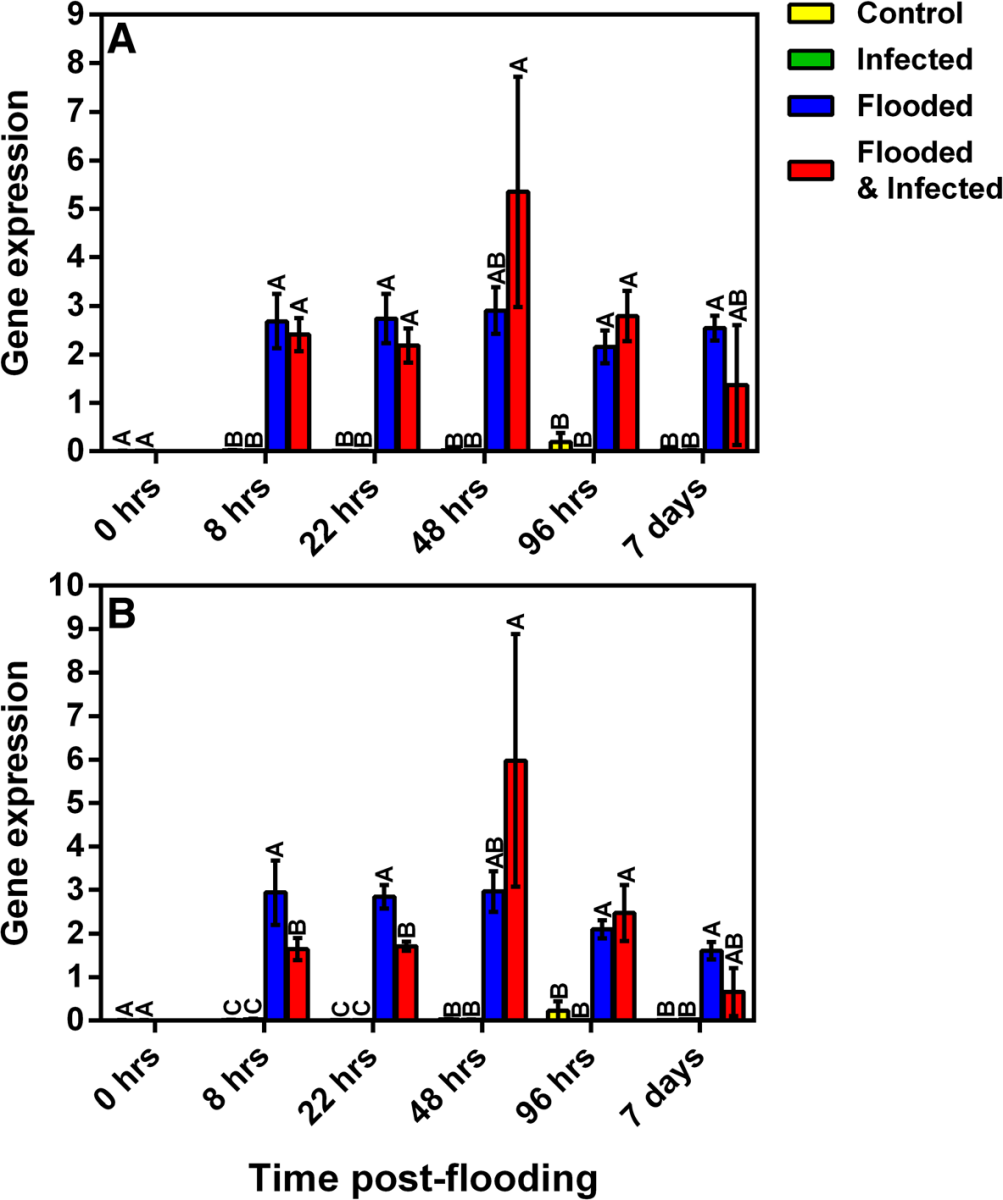
Time-course analysis of the relative gene expression of genes induced in avocado in response to flooding. Relative expression of *alcohol dehydrogenase b* (SinGI32N0T02IUGTU; **a**) and *pyruvate decarboxylase* (Contig 00088; **b**) Data were analysed using ANOVA and LS Means student’s *t*-test. Bars represented with the same letter are not significantly different at *P* < 0.05. Error bars indicate the standard error of the means (SEM) for three biological replicates, experiments were performed in triplicate. The x-axis represents the time after flooding was commenced

Glycolysis is followed by anaerobic fermentation under hypoxic conditions and induction of fermentation occurs in all species regardless of tolerance to flooding [11]. Fermentation of pyruvate to lactic acid by lactate dehydrogenase (LDH) generally precedes alcoholic fermentation via pyruvate decarboxylase (PDC) and alcohol dehydrogenase (ADH) [9, 19]. However, this leads to acidification of the cytoplasm and stimulates ADH whilst inhibiting LDH activity [9, 19]. No increases in transcripts representing LDH were seen within our dataset. The induction of LDH may occur during the early stages of hypoxia and may have already declined by 22 hpf, similar to poplar where levels of LDH increased after 5 h of flooding and declined thereafter [18]. Increased expression of *ADH* in avocado 8 hpf (Fig. 4a) suggests alcohol fermentation had already been induced by this time. Alcohol fermentation of pyruvate to yield ethanol occurs via PDC and ADH with acetaldehyde as an intermediate. NAD^+^ is regenerated and a further two molecules of ATP are produced through anaerobic fermentation and glycolytic reactions are sustained. *ADH*, *PDC*, and *pyrophosphate*-*fructose*-*6*-*phosphate 1*-*phosphotransferase* were among the most highly induced transcripts common to all flooding treatments in this study. *ADH* had increases in expression exceeding 40-fold at both 22 and 48 hpf. Expression of *PDC* (Contig 00088) was induced in flooded treatments as early as 8 hpf (Fig. 4b). Additionally, flooded plants that were not infected had significantly higher expression of this transcript than flooded, infected plants (Fig. 4b). Increased expression of this transcript in flooded relative to non-flooded plants was maintained for the duration of the experiment. A similar induction of *ADH* and *PDC* was seen in Gray poplar exposed to flooding and the enzyme activities of the corresponding enzymes were also seen to be increased [18]. In addition, enrichment analysis on transcripts induced by flooding indicated that terms related to PDC were over-represented in comparisons at 22 and 48 h post-flooding (Additional file 5: Figure S2 and Additional file 6: Figure S3). The induction of genes related to fermentative metabolism in response to flooding is clearly evident in avocado, as has been noted for other species [40]. The coordinated expression of these glycolytic and fermentation-related enzymes in response to hypoxia may suggest that there is a common regulatory mechanism which regulates expression of these genes such as the anaerobic response element (ARE) in maize and *Arabidopsis* [9].

**Fig. 5:**
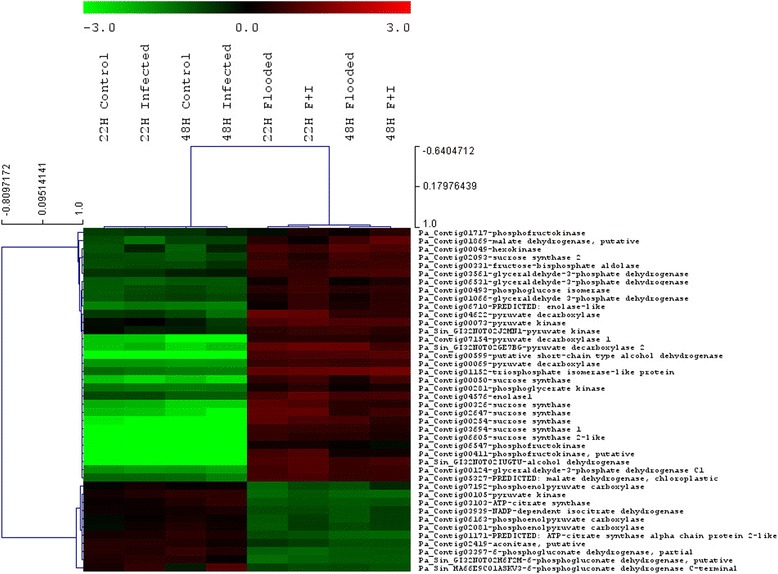
Expression of transcripts representing known anaerobic proteins differentially expressed in avocado in all flooded treatments when compared to non-flooded treatments at 22 and 48 hpf. Up-regulation of glycolytic and fermentation enzymes were seen across all flooded treatments. The colour-scale indicates log_2_FC and the branches of the trees are ordered according to the Pearson correlation coefficient (*r*)

Terms linked to sucrose synthase activity were found to be over-represented incomparisons of flooded to non-flooded treatments across both time-points. In addition, seven transcripts showing identity to sucrose synthase were clustered along with the glycolytic and citric acid cycle (TCA) enzymes and all seven were found to be induced in flooded treatments relative to non-flooded treatments (Fig. 5). The cleavage of sucrose yields glucose and fructose for use in glycolysis and is catalysed by invertases or sucrose synthases [8]. Under hypoxic conditions the cleavage of sucrose via sucrose synthase is thought to be favoured as less ATP is consumed during this reaction than when the reaction is catalysed by invertases [8, 11, 18]. Up-regulation of this transcript in response to flooding was confirmed by RT-qPCR (data not shown). This change in sucrose metabolism is probably related to increased demand for carbohydrates required to maintain glycolysis [18].

Transcripts showing homology to the enzymes involved in glycolysis, the TCA cycle, and the pentose phosphate pathway (PPP) were subsequently clustered to determine their expression patterns over the different conditions and time-points. Consistent with studies in rice, poplar, and *Arabidopsis* [11], decreased expression of transcripts belonging to the TCA and electron transport chain was seen in avocado (Fig. 5). These included two transcripts showing homology to *citrate synthase*, one assigned as *aconitase*, one as *isocitrate dehydrogenase*, and three representing *pyruvate carboxylase*. Only one transcript, identified as a putative *malate dehydrogenase*, showed increased expression in flooded treatments. The TCA cycle as well as electron transport chain form part of aerobic respiration and are responsible for yielding an additional 36 molecules of ATP [19]. There were three transcripts from the PPP that showed differential expression. All three corresponded to an enzyme from the oxidative part of the PPP, 6-phosphogluconate dehydrogenase, and showed decreased expression in flooded treatments (Fig. 5).

The 30 most induced and 30 most repressed transcripts differentially regulated in response to flooding were selected and filtered to identify highly regulated transcripts common between treatments (Tables 3 and 4). These transcripts were present at either 22 or 48 hpf, or at both time-points. Twenty-seven transcripts were found to be induced in all flooded treatments, of which 13 were induced at both time-points (Table 3). Seven of these 13 transcripts represented hypothetical, uncharacterized, or unknown proteins. Gene ontology classification of these commonly induced transcripts revealed that the largest proportions were represented by transport (13.4 %), catalytic activity (13.4 %), binding (9 %), transporter activity (7.5 %), hydrolase activity (6 %) and cell (6 %). *Alcohol dehydrogenase family protein* (Pa_Contig00297, Table 3) had an average fold-change of 41.19 (log_2_FC = 5.36) across the comparisons at 22 hpf and an average fold-change of 50.18 (log_2_FC = 5.64) at 48 hpf. Expression was not significantly different between the time-points.

**Table 3:** The avocado transcripts showing the greatest induction shared in all flooded to non-flooded comparisons at 22 and 48 h post-flooding

Seq ID	Annotation	e-value	GO process
Transcripts common between 22 and 48 h in the 30 most highly expressed transcripts			
Pa_Contig05239	*Unknown*		
Pa_Contig04056	*hypothetical protein PRUPE*_*ppa015169mg* [*Prunus persica*]	1e^-08^	
Pa_Contig00475	*PREDICTED*: *bidirectional sugar transporter SWEET1* [*Vitis vinifera*]	2.88E^-78^	GO:0016021,GO:0016020,GO:0008643, GO:0051119,GO:0006810,GO:0005783,GO:0005887,GO:0005886,GO:0034219
Pa_Contig00796	*cysteine desulfurylase*, *putative* [*Ricinus communis*]	2.08E^-76^	GO:0016740,GO:0008152,GO:0003824,GO:0031071,GO:0030170
Pa_Contig00929	*PREDICTED*: *7*-*alpha*-*hydroxysteroid dehydrogenase* [*Vitis vinifera*]	2.68E^-99^	GO:0005777
Pa_Contig03277	*PREDICTED*: *uncharacterized protein LOC100260129* [*Vitis vinifera*]	1.42E^-48^	GO:0005509
Pa_Contig06216	*hypothetical protein PRUPE*_*ppa013087mg* [*Prunus persica*]	8.74E^-19^	GO:0005509
Pa_Contig04134	*PREDICTED*: *uncharacterized protein LOC104223251* [*Nicotiana sylvestris*]	9e^-20^	
Pa_Contig07270	*Unknown*		
Pa_Contig00297	*alcohol*-*dehydrogenase family protein* [*Populus trichocarpa*]	0	GO:0046872,GO:0055114,GO:0016491,GO:0008270
Pa_Contig04583	*Unknown*		
Pa_Contig05040	*PREDICTED*: *2*-*aminoethanethiol dioxygenase*-*like* [*Glycine max*]	2.84E^-30^	GO:0055114,GO:0047800
Pa_Contig03479	*PREDICTED*: *ABC transporter B family member 21*-*like* [*Solanum tuberosum*]	8.63E^-88^	GO:0006855,GO:0009506,GO:0006200,GO:0005524,GO:0009735,GO:0009733,GO:0016021,GO:0008152,GO:0048767,GO:0055085,GO:0008559,GO:0000166,GO:0016887,GO:0010315,GO:0017111,GO:0042908,GO:0042626,GO:0006810,GO:0009630,GO:0010540,GO:0005886,GO:0010329,GO:0010328,GO:0060919
Transcripts common in all treatments present only at 22 h			
Pa_Contig04226	*2*-*nonaprenyl*-*3*-*methyl*-*6*-*methoxy*-*1*,*4*-*benzoquinol hydroxylase* [*Theobroma cacao*]	3e^-21^	
Pa_Contig07556	*PREDICTED*: *pyrophosphate*--*fructose 6*-*phosphate 1*-*phosphotransferase subunit alpha* [*Phoenix dactylifera*]	2e^-10^	
Pa_Sin_GI32N0T02IUGTU	*alcohol dehydrogenase* [*Citrus x paradisi*]	4.76E^-17^	GO:0046872,GO:0055114,GO:0016491,GO:0008270
Pa_NA_RC_Contig03244	*Unknown*		
Pa_Contig02574	*PREDICTED*: *universal stress protein MJ0531*-*like isoform 2* [*Solanum lycopersicum*]	3.11E^-60^	GO:0006950,GO:0005773
Pa_Contig00088	*pyruvate decarboxylase* [*Prunus armeniaca*]	0	GO:0030976,GO:0000287,GO:0008152,GO:0004737,GO:0003824,GO:0016829
Pa_Contig00434	*hypothetical protein PRUPE*_*ppa007712mg* [*Prunus persica*]	1.22E^-79^	GO:0003950,GO:0008152
Pa_Contig07234	*Unknown*		
Pa_Contig03497	*2*-*nonaprenyl*-*3*-*methyl*-*6*-*methoxy*-*1*,*4*-*benzoquinol hydroxylase* [*Theobroma cacao*]	2e^-19^	
Transcripts common in all treatments present only at 48 h			
Pa_Contig01112	*hypothetical protein OsI*_*03610* [*Oryza sativa Indica Group*]	9.52E^-104^	GO:0008152,GO:0003824,GO:0030170
Pa_Contig00627	*PREDICTED*: *mannose*-*specific lectin 3*-*like* [*Musa acuminata subsp. malaccensis*]	1e-26	
Pa_Contig01979	*hypothetical protein VITISV*_*003190* [*Vitis vinifera*]	7e^-15^	
Pa_Contig06346	*multidrug resistance protein 1*, *2*, *putative* [*Ricinus communis*]	4.55E^-28^	GO:0042626,GO:0016021,GO:0042908,GO:0016787,GO:0006810,GO:0006200,GO:0000166,GO:0008559,GO:0017111,GO:0015415,GO:0005524,GO:0016887,GO:0035435,GO:0006855,GO:0055085,GO:0008152
Pa_Contig03685	*conserved hypothetical protein* [*Ricinus communis*]	5.73E^-16^	GO:0016021,GO:0016020

Transcripts were chosen and filtered based on whether they were in the 30 most induced transcripts within all treatments

**Table 4:** The avocado transcripts showing the greatest repression shared in all flooded to non-flooded comparisons at 22 and 48 h post-flooding

Seq ID	Annotation	e-value	GO process
Transcripts common between 22 and 48 h in the 30 most highly repressed transcripts			
Pa_Contig01489	*Aquaporin PIP2.1*, *putative* [*Ricinus communis*]	2.12E^-78^	GO:0016021,GO:0016020,GO:0006810,GO:0005215
Pa_Contig01574	*TIP protein* [*Solanum lycopersicum*]	9.83E^-117^	GO:0072489,GO:0005215,GO:0042807,GO:0016021,GO:0016020,GO:0006810, GO:0009705,GO:0015200,GO:0009507
Pa_NA_F_contig07500	*Unknown*		
Pa_Contig05105	*subtilisin*-*like protease* [*Nicotiana tabacum*]	3.25E^-58^	GO:0006508,GO:0004252,GO:0008236,GO:0016787,GO:0043086,GO:0008233,GO:0042802
Pa_Contig00100	*GDP*-*L*-*galactose phosphorylase* [*Actinidia eriantha*]	1.68E^-163^	GO:0016779,GO:0016740,GO:0008152
Pa_Contig06179	*dihydroflavonol 4*-*reductase* [*Epimedium sagittatum*]	6.45E^-30^	GO:0044237,GO:0003824,GO:0050662
Pa_Contig00967	*phenylalanine ammonia*-*lyase* [*Cinnamomum osmophloeum*]	3.33E^-179^	GO:0009698,GO:0009058,GO:0016841,GO:0045548,GO:0005737,GO:0016829,GO:0003824,GO:0006559,GO:0009800
Pa_Contig01429	*dihydroflavinol reductase* [*Dendrobium moniliforme*]	3.18E^-63^	GO:0044237,GO:0003824,GO:0050662
Pa_Contig04550	*1*,*4*-*alpha*-*glucan*-*branching enzyme* [*Solanum tuberosum*]	1.18E^-76^	GO:0016740,GO:0043169,GO:0009501,GO:0016757,GO:0005978,GO:0005975,GO:0003824, GO:0019252,GO:0004553,GO:0003844,GO:0009507,GO:0009536
Pa_Contig07582	*PREDICTED*: *uncharacterized protein LOC100833771* [*Brachypodium distachyon*]	1e^-06^	
Transcripts present in all treatments present only at 22 h			
Pa_Contig03065	*PREDICTED*: *protein GAST1*-*like* [*Malus domestica*]	6e^-46^	
Pa_Contig07665	*chalcone synthase* [*Vitis vinifera*]	6.80E^-11^	GO:0009753,GO:0009926,GO:0009058,GO:0016210,GO:0009611,GO:0005634,GO:0009813,GO:0006979, GO:0009705,GO:0031540,GO:0009629,GO:0009733,GO:0005783,GO:0010224,GO:0008152,GO:0003824, GO:0016747,GO:0016746,GO:0016740
Pa_Contig03403	*Nucleobase ascorbate transporter* [*Medicago truncatula*]	5.44E^-85^	GO:0009506,GO:0016020,GO:0055085,GO:0006810,GO:0005215
Pa_Contig00312	*PREDICTED*: *homeobox*-*leucine zipper protein ATHB*-*6*-*like* [*Vitis vinifera*]	2.29E^-82^	GO:0009637,GO:0043565,GO:0042803,GO:0003700,GO:0003677,GO:0005634,GO:0045893,GO:0009737, GO:0009414,GO:0030308,GO:0009788,GO:0048573,GO:0006355,GO:0000976,GO:0048510,GO:0006351
Pa_NA_RC_Contig07454	*Unknown*		
Pa_Sin_FZ03KKT01A7ZOH	*PREDICTED*: *peroxidase 15*-*like* [*Solanum lycopersicum*]	8.70E^-27^	GO:0046872,GO:0055114,GO:0020037,GO:0016491,GO:0006979,GO:0004601
Pa_Contig00110	*3*-*deoxy*-*D*-*arabino*-*heptulosonate 7*-*phosphate synthase* [*Morinda citrifolia*]	0	GO:0003849,GO:0016829,GO:0009073
Pa_Contig00619	*chalcone synthase* [*Actinidia chinensis*]	5.71E^-167^	GO:0009058,GO:0016740,GO:0008152,GO:0003824,GO:0016747,GO:0016746
Pa_Contig02129	*glutathione S*-*transferase* [*Gossypium hirsutum*]	2.54E^-72^	GO:0016740,GO:0008152
Pa_Contig00126	*trans*-*cinnamate 4*-*hydroxylase* [*Populus tremuloides*]	0	GO:0046872,GO:0055114,GO:0016705,GO:0020037,GO:0016491,GO:0005506,GO:0004497
Transcripts present in all treatments present only at 48 h			
Pa_Contig01220	*aquaporin TIP1* [*Quercus petraea*]	1.50E^-111^	GO:0016021,GO:0016020,GO:0006810,GO:0005215
Pa_Contig05744	*chalcone synthase* [*Persea americana*]	6.02E^-60^	GO:0009058,GO:0016740,GO:0008152,GO:0003824,GO:0016746
Pa_NA_F_contig06354	*Unknown*		
Pa_Contig04602	*starch branching enzyme* [*Phaseolus vulgaris*]	8.55E^-51^	GO:0004553,GO:0005975,GO:0043169,GO:0003824
Pa_Contig01208	*glutathione S*-*transferase* [*Salicornia brachiata*]	5.75E^-79^	GO:0016740,GO:0008152
Pa_Contig01583	*asparagine synthetase family protein* [*Populus trichocarpa*]	2.32E^-102^	GO:0005524,GO:0070981,GO:0016874,GO:0006529,GO:0008152,GO:0000166,GO:0008652,GO:0004066

Transcripts were chosen and filtered based on whether they were in the top 30 most repressed transcripts within all treatments

There were nine transcripts that were commonly induced in all comparisons at 22 hpf (Table 3). Three of these represented unknown or hypothetical proteins. Interestingly, both *pyruvate decarboxylase* (Pa_Contig00088) and *alcohol dehydrogenase* (Pa_Sin_GI32N0T02IUGTU) were strongly induced in flooded treatments at this point. *Pyruvate decarboxylase* showed an average log_2_FC of 4.89 across the comparisons, corresponding to a fold-change of 29.55. At 48 h expression of this transcript was still significantly induced (Average log_2_FC = 4.83, fold-change = 28.49). Similarly, *alcohol dehydrogenase* (Pa_Sin_GI32N0T02IUGTU) showed an average increase in expression of 29.11 fold-change (log_2_FC = 4.86) at 22 hpf and 34.51 fold-change (log_2_FC = 5.10) at 48 hpf. Both pyruvate decarboxylase and alcohol dehydrogenase are involved in the alcoholic fermentation of pyruvate. A *PREDICTED*: *pyrophosphate*-*fructose 6*-*phosphate 1*-*phosphotransferase subunit alpha* (Pa_Contig07556) was found in all comparisons at 22 h (Table 3) and was found to have an average increase in expression of 27.19 fold-change (log_2_FC = 4.76) at 22 hpf and 17.67 fold-change (log_2_FC = 4.13) at 48 hpf. There was a significant decrease in expression from 22 to 48 hpf (fold-change = -2.00, log_2_FC = -0.99) in the FI treatment. The three main categories represented by the GO terms in this group were catalytic activity (29.4 %), binding (23.5 %) and metabolism (11.8 %).

**Fig. 6:**
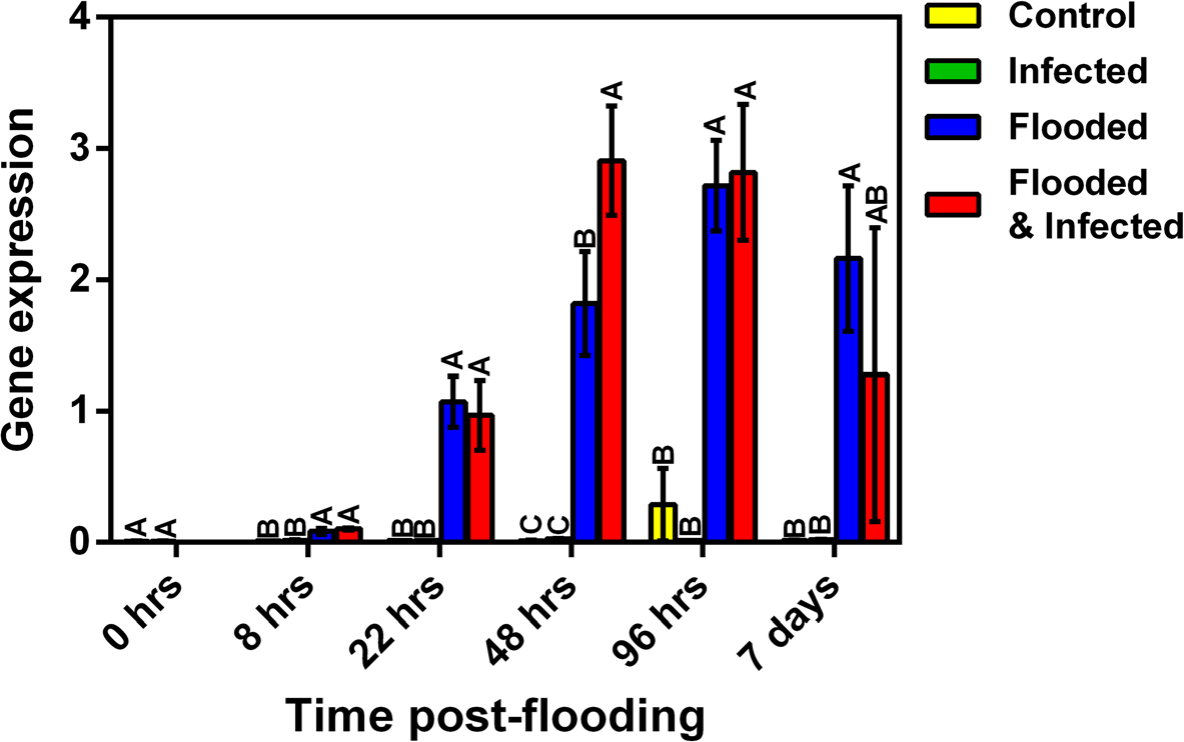
Time-course analysis of the relative gene expression of *multidrug resistance protein 1*, *2* (Contig 06346) expressed in avocado in response to flooding and infection by *P. cinnamomi*. Data were analysed using ANOVA and LS Means student’s *t*-test. Bars represented with the same letter are not significantly different at *P* < 0.05. Error bars indicate the SEM for three biological replicates, experiments were performed in triplicate. The x-axis represents the time after flooding was commenced

Five transcripts were among the most induced transcripts shared between comparisons at 48 hpf, three of which represented unknown or hypothetical proteins (Table 3). The other two transcripts corresponded to a *putative multidrug resistance protein* (Pa_Contig06346) and a *PREDICTED*: *mannose*-*specific lectin 3*-*like protein* (Pa_Contig00627). Pa_Contig06346 had an average increased fold-change of 17.67 (log_2_FC = 4.13) at this time-point for all flooded to non-flooded comparisons. Expression of this transcript was also increased at the earlier time-point (Average fold-change = 27.19, log_2_FC = 4.76). Multidrug resistance proteins (MRPs) have several roles in plants, including detoxification, stomatal regulation [41, 42] and are thought to play a role in the sequestration and exclusion from the cytoplasm of reactive metabolites which may cause cellular damage. Increased expression of *multidrug resistance protein 1*, *2* (06346) was apparent at 8 h in flooded treatments (Fig. 6). Differences in expression of this transcript became greater at 22 h and peaked at 48 h when flooded treatments were compared to non-flooded treatments. At 48 h there were also differences between the flooded avocados that were inoculated with *P. cinnamomi* and those that were not, with inoculated plants showing the highest expression. This difference was no longer apparent at 96 h where levels of expression were similar in all flooded treatments (Fig. 6). The trend in expression was maintained until 7 days when differences were no longer significant. The transcript annotated as *mannose*-*specific lectin 3*-*like protein* (Pa_Contig00627) showed major increases in expression (Average fold-change = 51.85, log_2_FC = 5.66) in the flooded treatments in comparison to the non-flooded treatments at 48 hpf. Although this transcript also showed increased expression (Average fold-change = 11.91, log_2_FC = 3.54) at 22 hpf, expression seemed to peak at the later time-point. Mannose-specific lectin proteins are thought to be involved in the recognition of foreign microorganisms through recognition of mannose type glycans [43].

### Inhibition of Energy Expenditure

Processes using excessive energy, such as cell-wall biosynthesis and lignin production decrease in plants exposed to hypoxia [18]. Terms relating to vacuole, apoplast, cell wall, and plasmodesmata were all found to be enriched in down-regulated transcripts. Down-regulation of defence-related transcripts was also seen in response to flooding (Table 4). Defence-related transcripts such as *chalcone synthase* (Pa_Contig and Pa_Contig00619), *peroxidase* (Pa_Sin_FZ03KKT01A7ZOH), and *glutathione S*-*transferase* (Pa_Contig02129) were amongst these. Some of these defence-related transcripts are involved in ROS production in response to pathogen invasion. This includes *peroxidases* [44], which were found to be highly repressed in response to flooding in avocado. In addition, defence-related transcripts also showed significant reductions at 48 h, with *chalcone synthase* (Pa_Contig05744, Average fold-change = -13.27, log_2_FC = -3.70) and *glutathione S*-*transferase* (Pa_Contig01208, Average fold-change = -11.42, log_2_FC = -3.51) being significantly repressed at this time-point. Induction of *chalcone synthase* has been seen in response to stress conditions such as UV light, bacterial or fungal infection and is a key enzyme in the flavonoid biosynthesis pathway [45]. Expression of *chalcone synthase* results in the accumulation of phytoalexins and has a role in the salicyclic acid defence pathway [45]. *Phenylalanine ammonia*-*lyase* (*PAL*, average fold-change = -11.90, log_2_FC = -3.55 at 22 h and average fold-change = -11.88, log_2_FC = -3.56 at 48 h) was one of the most repressed transcripts in avocado in all flooded treatments. PAL is involved in biosynthesis of flavonoids, phenylpropanoids and lignin and is the first step in the phenylpropanoid pathway [46]. This enzyme is induced in response to wounding, pathogens, temperatures and several other external stimuli [46]. This may indicate that energy usually allocated to defence responses is redirected to other processes that are more important to survival under flooding conditions, which may account for the increased susceptibility to pathogens, in particular root rots, often associated with flooded conditions. Other processes that were down-regulated in flooded treatments represent processes not prioritized under these conditions of stress, such as the response to cadmium, an environmental pollutant, response to salt, hormone stimulus, response to water deprivation and response to temperature stimulus. Additionally, several transcripts showing significant repression represented sequences that were unknown or hypothetical proteins (Table 4).

### Role of Aquaporins in Flooding Response

**Fig. 7:**
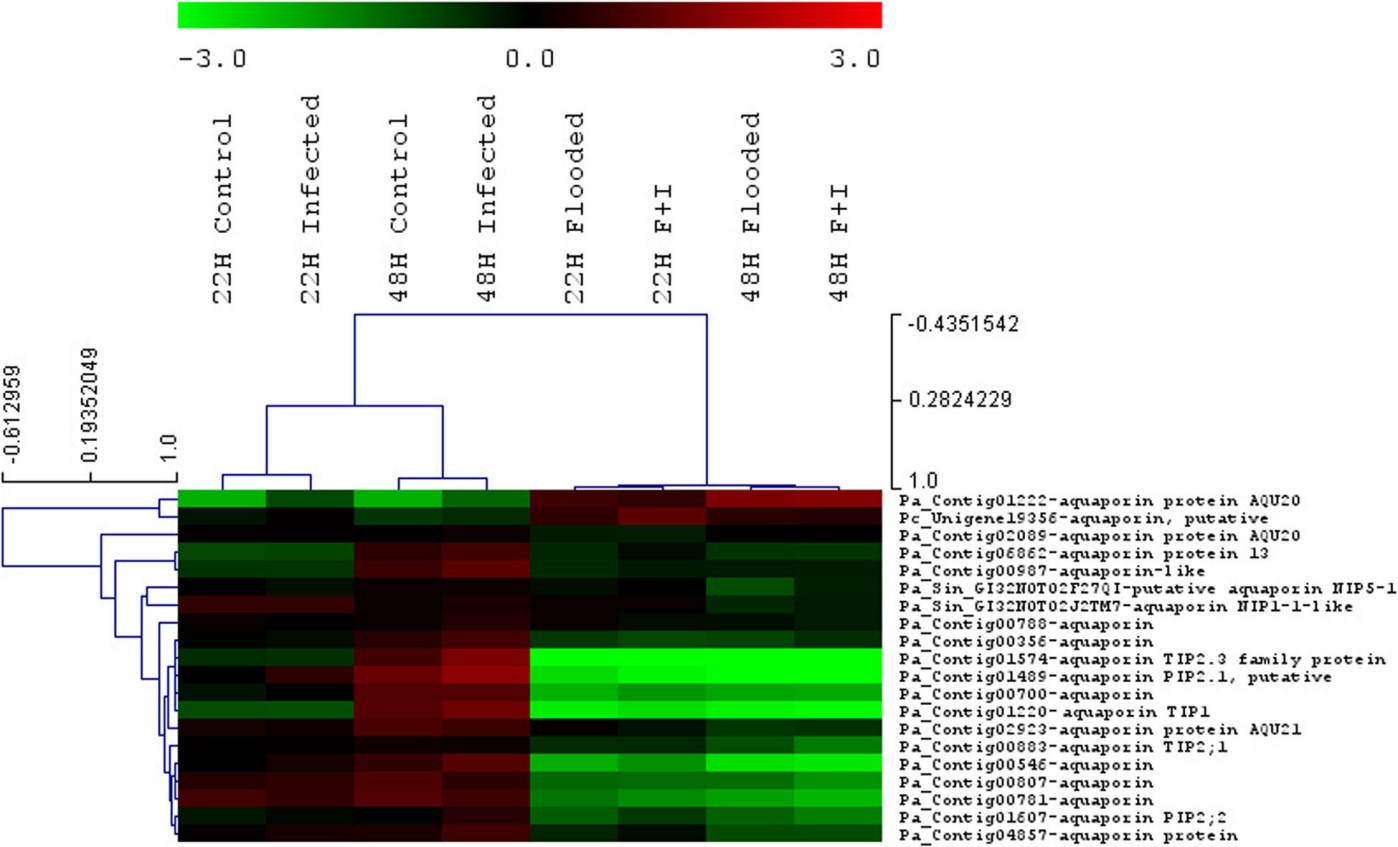
The decrease in aquaporin expression observed in avocado exposed to flooding and *P. cinnamomi* infection. The colour-scale indicates log_2_FC and the branches of the trees are ordered according to the Pearson correlation coefficient (*r*)

Aquaporins are water channel proteins that belong to the plasma membrane intrinsic protein (PIP) family and contribute to the regulation of root hydraulic conductivity in *Arabidopsis* [17]. Expression of aquaporin genes can be perturbed by abiotic stress [47] and flooding has been seen to lead to reduced expression in some trees [18, 39]. Enrichment analysis of avocado transcripts that were down-regulated indicated that at both 22 hpf as well as 48 hpf the majority of sequences were associated with plasma membrane (GO:0005886) in flooded treatments. Several aquaporin family proteins were also seen to be amongst the top most repressed transcripts (Table 4). *Aquaporin PIP2.1* (Pa_Contig01489, average fold-change = -8.23, log_2_FC = -3.01 at 22 h and average fold-change = -24.36, log_2_FC = -4.59 at 48 h) and *TIP protein* (Pa_Contig01574, average fold-change = -7.64, log_2_FC = -2.93) at 22 h and average fold-change = -32.60, log_2_FC = -4.97 at 48 h) were amongst the 30 most repressed transcripts for both 22 hpf as well as 48 hpf. An additional aquaporin, *TIP1* (Pa_Contig01220, average fold-change = -3.42, log_2_FC = -1.77 at 22 h and average fold-change = -16.88, log_2_FC = -4.07 at 48 h) was found to be amongst the transcripts showing the greatest repression at 48 h. This transcript was also repressed at the 22 h time-point suggesting that onset of repression occurs relatively early. The expression of all 20 transcripts showing homology to aquaporins was investigated and most were either not expressed or showed reductions in expression in response to flooding (Fig. 7). Only one avocado aquaporin showed increased expression. Previous studies have noted that different aquaporins can show opposite trends in gene expression in response to a particular stress. We suggest that aquaporins are important in the regulation of root hydraulic conductivity in avocado under flooded conditions. Reduced root hydraulic conductivity can ultimately lead to stomatal closure in avocado, which has previously been seen to occur in avocado in response to flooding [48].

**Fig. 8:**
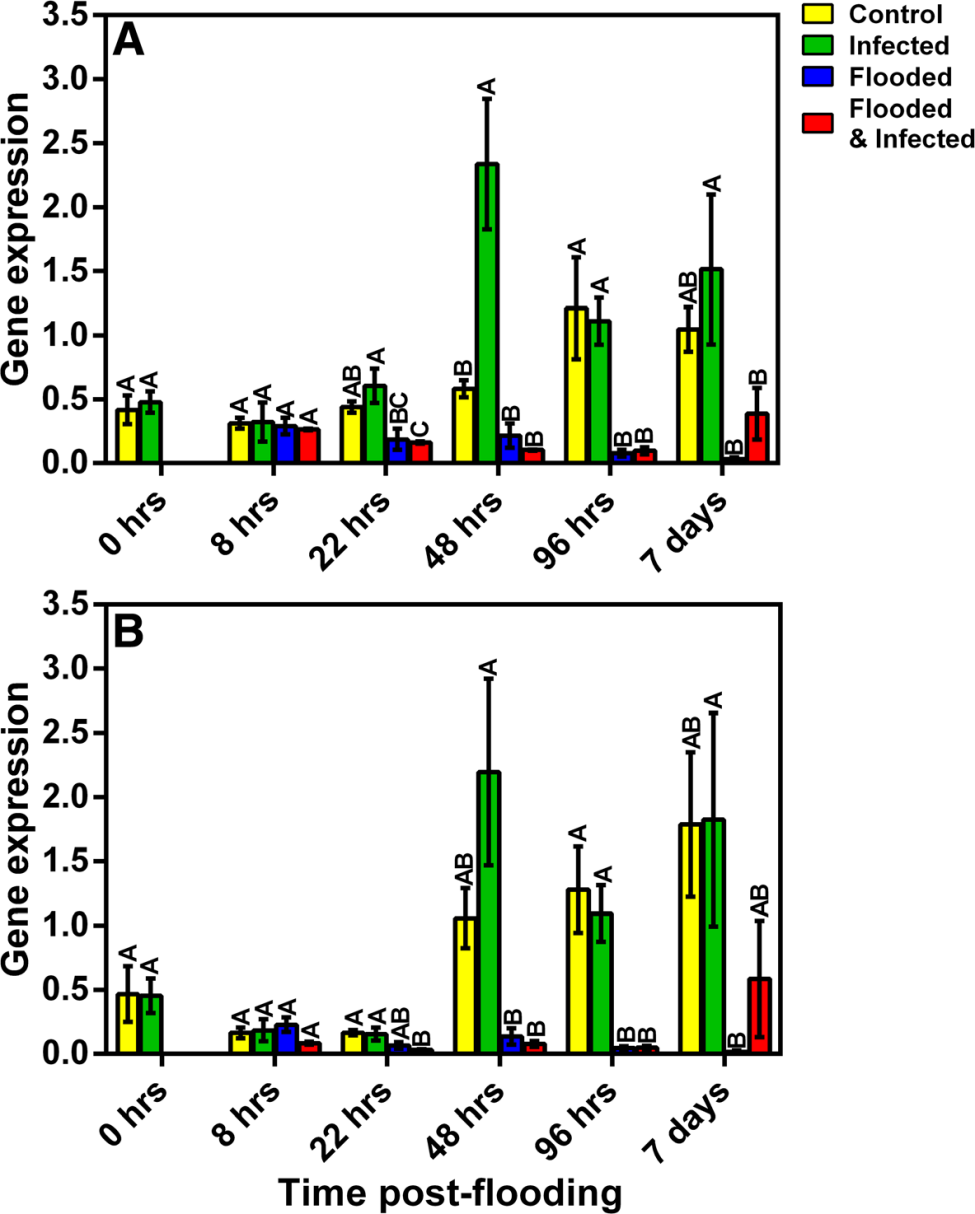
Time-course analysis of the relative gene expression of two avocado aquaporins. Relative expression of *plasma membrane intrinsic protein* (00546; **a**) and *membrane channel protein* (01220; **b**) over six time-points is shown. Data were analysed using ANOVA and LS Means student’s *t*-test. Bars represented with the same letter are not significantly different at *P* < 0.05. Error bars indicate the SEM for three biological replicates, experiments were performed in triplicate. The x-axis represents the time after flooding was commenced

The expression of two aquaporins was investigated further using qRT-PCR and assessing expression over an extended time-course (Fig. 8). Expression of *plasma membrane intrinsic protein* (Contig 00546) was adversely affected by flooding, exhibiting reduced levels in these treatments relative to non-flooded treatments. Reduced levels of this transcript were seen in plants that were flooded at 22 hpf, with plants that were exposed to the combination of stresses showing significant reductions in expression (Fig. 8a). Interestingly, plants that were infected in the absence of flooding showed increased levels of expression relative to flooded treatments at 22 hpf and by 48 hpf levels were significantly increased relative to all treatments. Flooded treatments exhibited significantly decreased expression of this aquaporin by 96 hpf (Fig. 8a). This trend was maintained at 7 days post-flooding, although infected plants once again demonstrated slightly higher expression levels than control plants and significantly higher levels than that observed in the flooded treatments. Similarly to contig 00546, reductions in expression of *membrane channel protein* (contig 01220) were induced 22 hpf, with significant reductions seen in the combination treatment (Fig. 8b). Once again expression of the aquaporin was highest in infected plants by 48 hpf. Non-flooded treatments showed higher expression of transcript contig 01220 than both flooded treatments by 96 hpf (Fig. 8b). Expression of contig 01220 in the combination treatment showed similar increases in expression as that seen in contig 00546 by 7 days post-flooding. Transcript levels in the flooding treatment remained low (Fig. 8). The increased expression of two different aquaporins in response to infection is interesting, suggesting that this may serve to alter hydraulic conductivity as a general stress response.

## Conclusions

This study represents the first large-scale gene expression analysis assessing the response of avocado to flooding and *P. cinnamomi*. Unravelling the molecular mechanisms that are involved in the response of plants to flooding and generating a comprehensive model of this response is challenging. However, this will enable the elucidation of patterns of plant distribution and abundance in natural flood-prone environments and aid in the selection and development of crops with improved flooding tolerance. The focus of this study was to determine the impact of flooding on avocado and to determine whether this was affected by the presence of *P. cinnamomi*. In addition, identification of genes integral to the response to flooding by avocado is important in order to develop rootstocks that display tolerance to flood conditions. Flooding induced large transcriptomic changes in avocado regardless of whether plants were infected with *P. cinnamomi* or not. This is likely explained by the large metabolic disruptions caused by flooding which masks the more subtle responses to the pathogen. However, many of the genes affected in flooded plants are defence-related transcripts. These transcripts were generally repressed in flooded conditions in order to limit energy expenditure under the O_2_-limited conditions caused by flooding. This may contribute to the increased susceptibility of flooded or water-logged avocado to *P. cinnamomi*. Transcripts encoding glycolytic enzymes, enzymes involved in fermentation, and transcripts related to sucrose metabolism were induced in flooded treatments. This illustrates that maintenance of energy-balance is important in avocado under these conditions. Aquaporins were found to be strongly down-regulated by the imposition of flooding and this may explain the reductions seen in stomatal conductance in avocado in response to flooding [48]. These reductions occur as aquaporins are important in root hydraulic conductivity, which can ultimately affect stomatal function. A large proportion of the genes that were significantly affected by flooding in avocado either had no homology to known sequences or represented hypothetical or predicted proteins. This has been noted in previous studies and will require the selection of candidate genes for functional annotation. Clustering of these transcripts with transcripts of known function may aid in the functional characterization of these unknown genes. In *Arabidopsis*, mutation of several *HUP proteins* caused mutants to display significantly altered tolerance to submergence and indicates that these poorly characterized proteins may contain a wealth of candidates for manipulation of the response of plants to hypoxia [49]. Our study has identified numerous genes with no defined function that can possibly contribute to tolerance to flooding in avocado and will require further study. It is important to note that spatial information, such as how genes induced at the same time-point relate to one another is not necessarily resolved by microarray analysis and further studies will be needed to elucidate this.

## Additional files

Additional file 1: Table S1. Primers used in the RT-qPCR validation of the microarray data. The putative identities assigned to each transcript are listed in the ‘Gene’ column. (DOCX 22 kb) Additional file 2: Table S2. RT-qPCR validation of microarray data. Representative arrays chosen for microarray validation. Five transcripts were selected to ensure the microarray data was comparable with other expression profiling methods. Values indicate fold-changes in gene expression. (DOCX 21 kb) Additional file 3: Table S3. Avocado transcripts found to be up-regulated in the infected treatment (I) compared to the control treatment (C) at 48 h-post flooding (8 days post-infection). (DOCX 22 kb) Additional file 4: Figure S1. Comparison of the repressed avocado transcripts in flooded to non-flooded treatments at 22 h post-flooding (A) and 48 h post-flooding (B). Values for transcripts with more than one probe present on the array were first averaged and then subjected to the thresholds to determine differential expression. (TIF 3090 kb) Additional file 5: Figure S2. Differential GO-term distribution after enrichment analysis for sequences up-regulated in the 22HF vs. 22HI comparison. The percentages of sequences associated with GO terms showing over-representation in the 22HF vs. 22HI comparison compared to the reference set consisting of all sequences on the array (FDR < 0.05). Only transcripts showing significant differential expression (log_2_FC > 1, adj. *P*-value < 0.05) were included in the analysis. (PNG 96 kb) Additional file 6: Figure S3. Differential GO-term distribution after enrichment analysis for sequences up-regulated in the 48HFI vs. 48HC comparison. The percentages of sequences associated with GO terms showing over-representation in the 48HFI vs. 48HC comparison compared to the reference set consisting of all sequences on the array (FDR < 0.05). Only transcripts showing significant differential expression (log_2_FC > 1, adj. *P*-value < 0.05) were included in the analysis. (PNG 110 kb)

## Abbreviations

ADH: Alcohol dehydrogenase
ARE: Anaerobic response element
B2G: Blast2GO software
C: Control plants
dCAS: Desktop cDNA Annotation System
F: Flooded plants
FC: Fold change
FDR: False discovery rate
FI: Plants exposed to a combination of flooding and infection
GAL: GenePix Array List
gDNA: Genomic DNA
HCL: Hierarchical clustering
hpf: Hours post-flooding
I: Infected plants
LDH: Lactate dehydrogenase
LIMMA: Linear models for microarray data
MeV: Multi Experiment Viewer
MRPs: Multidrug resistance proteins
PDC: Pyruvate decarboxylase
PIP: Plasma membrane intrinsic protein
PPP: Pentose phosphate pathway
PRR: Phytophthora root rot
ROS: Reactive oxygen species
SNR: Signal-to-noise ratios
TCA: Citric acid cycle
