# Supplementary material for: Transcriptome responses of an ungrafted Phytophthora root rot tolerant avocado (Persea americana) rootstock to flooding and Phytophthora cinnamomi
Source: BMC Plant Biol. 2016 Sep 22;16:205. doi: 10.1186/s12870-016-0893-2 (PMC5034587; doi:10.1186/s12870-016-0893-2)
Supplement: Additional file 1: Table S1. — Primers used in the RT-qPCR validation of the microarray data. The putative identities assigned to each transcript are listed in the ‘Gene’ column. (DOCX 22 kb) [file 12870_2016_893_MOESM1_ESM.docx]

**Additional file 1: Table S1**

| **Gene** | **Seq ID** | **Direction** | **Sequence (5’-3’)** | **Annealing temp.** |
| --- | --- | --- | --- | --- |
| ***Actin*** | 00256 | Forward | CCAAGCAGCATGAAGATAAAGGT | 58°C |
|  |  | Reverse | CACATCTGTTGGAAGGTGCTC |  |
| ***18S*** | - | Forward | GTTACTTTAGGACTCCGCC | 58°C |
|  |  | Reverse | TTCCTTTAAGTTTCAGCCTTG |  |
| ***Alpha-1 tub*** | - | Forward | AAGGATTATGAGGAGGTTG | 56°C |
|  |  | Reverse | ATCAGCCACATTCTCTTC |  |
| ***Sucrose synthase*** | 00004 | Forward | CATACATCAAACCGTGAGATCCA | 57°C |
|  |  | Reverse | GTACTACTTGCAACCAGCGT |  |
| ***Non-symbiotic hemoglobin*** | 04393 | Forward | TCAAGATGACCTGTGAAGCA | 57°C |
|  |  | Reverse | CCTTCTTAAGATGAACTGAACCC |  |
| ***Pyruvate decarboxylase*** | 01547 | Forward | GAGGGTGCAAACACAATGGA | 58°C |
|  |  | Reverse | CGCAATACAGTAACCCAAACCA |  |
| ***Endochitinase*** |  | Forward | ATCACCAACATCATCAAC | 57°C |
|  |  | Reverse | CTCTTGTAGAAGCCAATG |  |
| ***Plasma membrane intrinsic protein*** | 00546 | Forward | TGGGTATACTAAGGGAGATGGG | 58°C |
|  |  | Reverse | CCAAGATAGGCACATGGGAG |  |
| ***Membrane channel protein*** | 01220 | Forward | CTACGGTGACATCTTCATTGG | 55°C |
|  |  | Reverse | ACAAATTAACAGGAGCAGCAG |  |
| ***Pyruvate decarboxylase, putative*** | 00088 | Forward | GCCTTACAATGTCATCAAGAACTG | 56°C |
|  |  | Reverse | CTCCTCTTCTGTCCTCACCT |  |
| ***Multidrug resistance protein 1, 2, putative*** | 06346 | Forward | AAGCAAAGACATCTACTGCCTC | 55°C |
|  |  | Reverse | GAGAAACTCAATGTTTCCCTTCAC |  |
| ***Alcohol dehydrogenase b*** | SinGI32N0T02IUGTU | Forward | TGGTTGCTGTGCTAGTAGGT | 60°C |
|  |  | Reverse | TTCCAAAGAAAGTTCCCTTGAGAG |  |
