# Supplementary material for: Transcriptome responses of an ungrafted Phytophthora root rot tolerant avocado (Persea americana) rootstock to flooding and Phytophthora cinnamomi
Source: BMC Plant Biol. 2016 Sep 22;16:205. doi: 10.1186/s12870-016-0893-2 (PMC5034587; doi:10.1186/s12870-016-0893-2)
Supplement: Additional file 2: Table S2. — RT-qPCR validation of microarray data. Representative arrays chosen for microarray validation. Five transcripts were selected to ensure the microarray data was comparable with other expression profiling methods. Values indicate fold-changes in gene expression. (DOCX 21 kb) [file 12870_2016_893_MOESM2_ESM.docx]

**Additional file 2 Table S2**

| **Gene** | **22HT3vsT2**  **Array RT-qPCR** | | **22HT3vsT1**  **Array RT-qPCR** | | **22HT4vsT3**  **Array RT-qPCR** | | **48HT3vsT1**  **Array RT-qPCR** | |
| --- | --- | --- | --- | --- | --- | --- | --- | --- |
| **00546** | 0.83 | 0.58 | 3.20 | 2.4 | 0.23 | 0.47 | 10.46 | 4.55 |
| **01220** | 1.02 | 0.99 | 3.23 | 4.37 | 0.27 | 0.47 | 15.53 | 13.67 |
| **00088** | 0.76 | 0.75 | 0.03 | 0.005 | 26.79 | 357.67 | 0.04 | 0.005 |
| **06346** | 1.02 | 0.86 | 0.03 | 0.01 | 23.68 | 90.56 | 0.01 | 0.002 |
| **Sin_G** | 1.07 | 1.06 | 0.03 | 0.003 | 27.16 | 371.70 | 0.03 | 0.002 |
