# Supplementary material for: Transcriptome responses of an ungrafted Phytophthora root rot tolerant avocado (Persea americana) rootstock to flooding and Phytophthora cinnamomi
Source: BMC Plant Biol. 2016 Sep 22;16:205. doi: 10.1186/s12870-016-0893-2 (PMC5034587; doi:10.1186/s12870-016-0893-2)
Supplement: Additional file 3: Table S3. — Avocado transcripts found to be up-regulated in the infected treatment (I) compared to the control treatment (C) at 48 h-post flooding (8 days post-infection). (DOCX 22 kb) [file 12870_2016_893_MOESM3_ESM.docx]

**Additional file 3: Table S3**

| **Sequence identifier** | **Annotation** | **e-value** | **GO term** |
| --- | --- | --- | --- |
| Pa_Contig06344 | serine carboxypeptidase, putative [Ricinus communis] | 3.98E-32 | GO:0006508,GO:0004180,GO:0016787,GO:0004185 |
| Pa_Sin_HA66E9C01ASKV3 | 6-phosphogluconate dehydrogenase C-terminal [Glarea lozoyensis ATCC 20868] | 7.65E-14 | GO:0055114,GO:0016491,GO:0006098,GO:0016616,GO:0004616,GO:0050662,GO:0050661 |
| Pa_Contig07667 | Cytochrome P450 | 7.51E-10 | GO:0020037,GO:0005506,GO:0016021,GO:0016705,GO:0016020,GO:0055114,GO:0016491,GO:0005789,GO:0009835,GO:0043231,GO:0046872,GO:0005783,GO:0031090,GO:0004497 |
| Pa_NA_RC_Contig07628 | Unknown |  |  |
| Pa_Contig00472 | PREDICTED: chitotriosidase-1 [Vitis vinifera] | 2.12E-121 | GO:0004568,GO:0009737,GO:0005975,GO:0008843,GO:0035885,GO:0009651,GO:0004553,GO:0009753,GO:0005618,GO:0006032 |
| Pa_Contig01063 | PREDICTED: pathogen-related protein-like [Prunus mume] | 8e-114 |  |
| Pa_Contig00520 | GDSL esterase/lipase At3g26430 isoform 1 [Glycine max] | 4.36E-124 | GO:0006629,GO:0016788,GO:0016298,GO:0016787 |
| Pa_Contig00542 | beta-1,3-glucanase [Vitis vinifera] | 1.13E-132 | GO:0005774,GO:0009817,GO:0016787,GO:0005975,GO:0009651,GO:0004553,GO:0008152,GO:0005618,GO:0002215,GO:0016798 |
| Pa_Contig05854 | PREDICTED: protein HOTHEAD-like [Musa acuminata subsp. malaccensis] | 4e-17 |  |
| Pa_Contig01236 | germin-like protein [Ananas comosus] | 1.18E-68 | GO:0046872,GO:0045735,GO:0030145,GO:0005576 |
| Pa_Contig06581 | hypothetical protein JCGZ_21815 [Jatropha curcas] | 4e-05 |  |
| Pa_Contig00205 | PREDICTED: protein HOTHEAD isoform 1 [Vitis vinifera] | 1.59E-171 | GO:0055114,GO:0008812,GO:0006066,GO:0016614,GO:0050660 |
| Pa_Contig05213 | protease inhibitor-like [Glycine max] | 2.54E-18 | GO:0006508,GO:0010951,GO:0008233,GO:0009611,GO:0004867 |
| Pa_Contig05072 | potato inhibitor I family protein [Populus alba x Populus glandulosa] | 4.60E-20 | GO:0010951,GO:0009611,GO:0004867 |
| Pa_Contig01014 | Class I chitinase [Picea engelmannii x Picea glauca] | 5.39E-110 | GO:0006032,GO:0008061,GO:0005975,GO:0004568,GO:0016998 |
| Pa_Contig06358 | hypothetical protein JCGZ_21815 [Jatropha curcas] | 2e-09 |  |
