# Supplementary figures and images for: Transcriptome responses of an ungrafted Phytophthora root rot tolerant avocado (Persea americana) rootstock to flooding and Phytophthora cinnamomi
Source: BMC Plant Biol. 2016 Sep 22;16:205. doi: 10.1186/s12870-016-0893-2 (PMC5034587; doi:10.1186/s12870-016-0893-2)

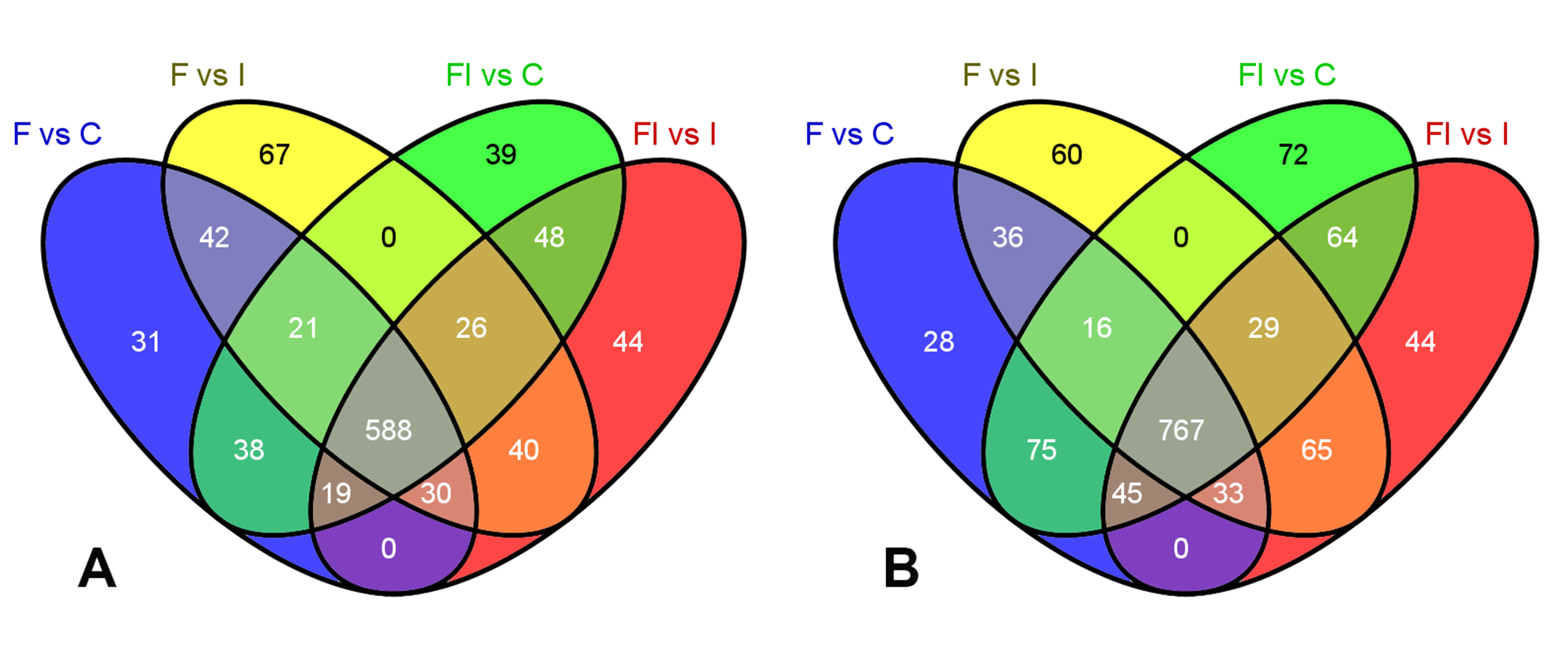

Supplement: Additional file 4: Figure S1. — Comparison of the repressed avocado transcripts in flooded to non-flooded treatments at 22 h post-flooding (A) and 48 h post-flooding (B). Values for transcripts with more than one probe present on the array were first averaged and then subjected to the thresholds to determine differential expression. (TIF 3090 kb) [file 12870_2016_893_MOESM4_ESM.tif]

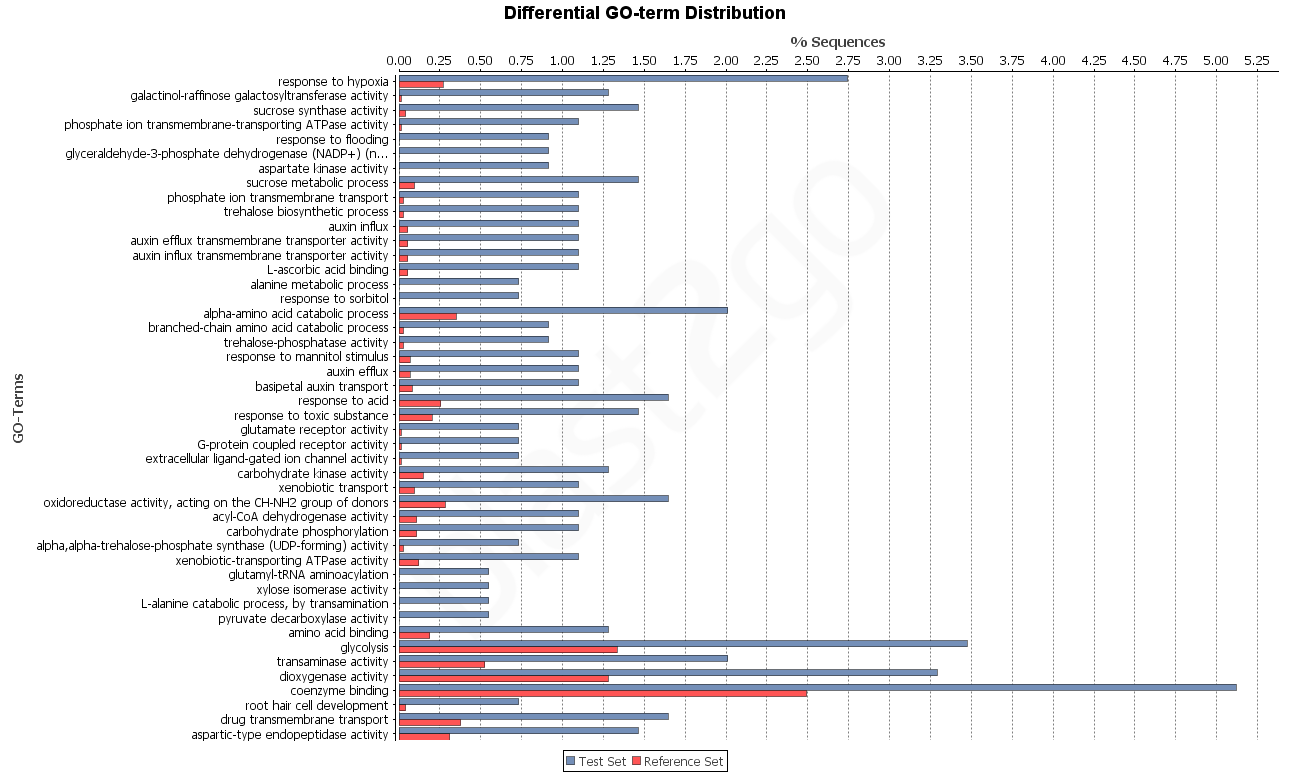

Supplement: Additional file 5: Figure S2. — Differential GO-term distribution after enrichment analysis for sequences up-regulated in the 22HF vs. 22HI comparison. The percentages of sequences associated with GO terms showing over-representation in the 22HF vs. 22HI comparison compared to the reference set consisting of all sequences on the array (FDR < 0.05). Only transcripts showing significant differential expression (log2FC > 1, adj. P-value < 0.05) were included in the analysis. (PNG 96 kb) [file 12870_2016_893_MOESM5_ESM.png]

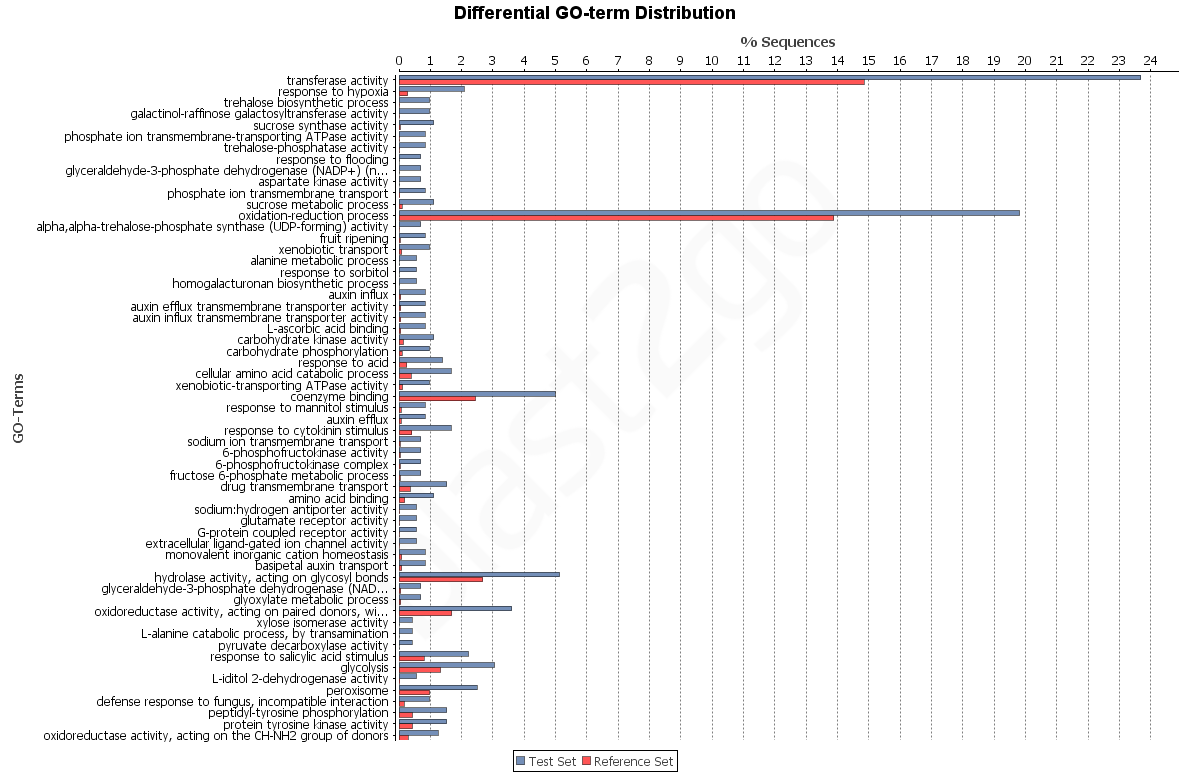

Supplement: Additional file 6: Figure S3. — Differential GO-term distribution after enrichment analysis for sequences up-regulated in the 48HFI vs. 48HC comparison. The percentages of sequences associated with GO terms showing over-representation in the 48HFI vs. 48HC comparison compared to the reference set consisting of all sequences on the array (FDR < 0.05). Only transcripts showing significant differential expression (log2FC > 1, adj. P-value < 0.05) were included in the analysis. (PNG 110 kb) [file 12870_2016_893_MOESM6_ESM.png]
